# Niche adaptation and viral transmission of human papillomaviruses from archaic hominins to modern humans

**DOI:** 10.1371/journal.ppat.1007352

**Published:** 2018-11-01

**Authors:** Zigui Chen, Rob DeSalle, Mark Schiffman, Rolando Herrero, Charles E. Wood, Julio C. Ruiz, Gary M. Clifford, Paul K. S. Chan, Robert D. Burk

**Affiliations:** 1 Departments of Microbiology, Faculty of Medicine, The Chinese University of Hong Kong, Hong Kong SAR, China; 2 Sackler Institute of Comparative Genomics, American Museum of Natural History, New York, NY, United States of America; 3 Division of Cancer Epidemiology and Genetics, National Cancer Institute, National Institutes of Health, Rockville, MD, United States of America; 4 International Agency for Research on Cancer, World Health Organization, Lyon, France; 5 Proyecto Epidemiológico Guanacaste, Fundación INCIENSA, San José, Costa Rica; 6 Department of Pathology, Wake Forest School of Medicine, Winston-Salem, NC, United States of America; 7 Department of Veterinary Sciences, The University of Texas MD Anderson Cancer Center, Bastrop, Texas, United States of America; 8 Departments of Pediatrics, Microbiology and Immunology; Epidemiology and Population Health; Obstetrics, Gynecology and Woman’s Health, Albert Einstein College of Medicine, Bronx, NY, United States of America; University of Utah, UNITED STATES

## Abstract

Recent discoveries on the origins of modern humans from multiple archaic hominin populations and the diversity of human papillomaviruses (HPVs) suggest a complex scenario of virus-host evolution. To evaluate the origin of HPV pathogenesis, we estimated the phylogeny, timing, and dispersal of HPV16 variants using a Bayesian Markov Chain Monte Carlo framework. To increase precision, we identified and characterized non-human primate papillomaviruses from New and Old World monkeys to set molecular clock models. We demonstrate specific host niche adaptation of primate papillomaviruses with subsequent coevolution with their primate hosts for at least 40 million years. Analyses of 212 HPV16 complete genomes and 3582 partial sequences estimated ancient divergence of HPV16 variants (between A and BCD lineages) from their most recent common ancestors around half a million years ago, roughly coinciding with the timing of the split between archaic Neanderthals and modern *Homo sapiens*, and nearly three times longer than divergence times of modern *Homo sapiens*. HPV16 A lineage variants were significantly underrepresented in present African populations, whereas the A sublineages were highly prevalent in European (A1-3) and Asian (A4) populations, indicative of viral sexual transmission from Neanderthals to modern non-African humans through multiple interbreeding events in the past 80 thousand years. Remarkably, the human leukocyte antigen B*07:02 and C*07:02 alleles associated with increased risk in cervix cancer represent introgressed regions from Neanderthals in present-day Eurasians. The archaic *hominin-host-switch* model was also supported by other HPV variants. Niche adaptation and virus-host codivergence appear to influence the pathogenesis of papillomaviruses.

## Author summary

Epidemiologic studies have demonstrated that persistent infection of select oncogenic human papillomaviruses (HPVs) is the main cause of cervix precancer and cancer. Nevertheless, our knowledge of the underlying evolutionary mechanisms driving the divergence and emergence of viral oncogenicity in specific types of HPVs is incomplete. To better understand the molecular evolution of oncogenic HPVs, we isolated viruses from non-human primates, evaluated papillomavirus molecular clock models, and estimated the divergence times of HPV16 and other HPV type variants from their most recent common ancestors. Primate PV-host tissue tropisms indicated niche adaptation of viruses to host ecosystems as the first stage of the evolution of oncogenic HPVs. The data also provided evidence of ancient codivergence of HPV variants with archaic hominins and recent viral transmission from Neanderthals to modern non-African humans through sexual intercourse. Understanding the evolution of papillomaviruses should provide important biological insights and suggest mechanisms underlying HPV-induced cervical cancer, since niche adaptation rather than oncogenicity drives viral fitness.

## Introduction

Papillomaviruses (PVs) are ubiquitous, non-enveloped, small double-stranded circular DNA viruses that cause proliferation of epithelial cells in a wide range of vertebrate host species, from reptiles to mammals [1, 2]. Currently, over 200 PVs infecting primate hosts (human and non-human) have been characterized and shown to group predominantly within 3 highly divergent genera—*Alphapapillomavirus*, *Betapapillomavirus*, and *Gammapapillomavirus* [3]. All oncogenic PVs associated with the development of cervical carcinoma, including human PV (HPV) types 16, 18, 31, 33, 35, 39, 45, 51, 52, 56, 58, and 59 and *Macaca fascicularis* PV type 3 (MfPV3), share a common ancestor within the *Alphapapillomavirus* [4–7]. Among these oncogenic types, which are sexually transmitted primarily through intercourse [8, 9], HPV16 is globally the most prevalent HPV type detected, suggesting an increased fitness [10–12]. Moreover, HPV16 is also the most common HPV type detected in cervical cancer, which is the fourth most common cancer among women worldwide [13]. Nevertheless, most exposures to HPV types are transient, and many PVs appear to be more commensal than pathogenic [14].

Strict coevolution of a host and its pathogen is more likely if the pathogen is transmitted vertically and there is little or no cross-species acquisition. Persistent infection by pathogens generally indicates that they are well adapted to their host and that extinction will be rare so long as the host survives. Hence, in scenarios of coevolution, the evolutionary history of a pathogen should mirror that of its host, both in divergence times and phylogenic history (Fahrenholz’s rule) [15, 16]. These criteria have been shown to hold for feline PVs within the genus *Lambdapapillomavirus* isolated from oral lesions [17]. On the other hand, horizontal transmission of pathogens through host switching without restricted species specificity will produce a very different evolutionary history between host and pathogen. In hosts harboring many different types of PVs (e.g., bovines, humans, and macaques), the selection pressure exerted by PVs on their hosts appears negligible in comparison with what the hosts exert on the PV pathogens. Within human populations, for example, the ancient dispersal of HPV variants (e.g., HPV16 and HPV58) challenges a simple evolutionary pattern of viruses migrating with modern *Homo sapiens* [18], and instead indicates codivergence of viruses with archaic hominins and transmission to modern humans [19, 20]. The genetic heterogeneity of PVs implies a complex evolutionary history with many interacting factors, including but not limited to virus-host codivergence, tissue tropism, lineage sorting, transmission, recombination, and natural selection [21, 22]. Understanding the capacity for, and history of, viral adaptation to host ecological environments is essential for understanding the genetic basis of HPV carcinogenicity [23]. However, the origin and evolution of oncogenic PVs remains poorly understood.

In this report, we estimate the divergence times of HPV16 and other oncogenic HPV types using a well-established Bayesian molecular clock model with newly characterized primate PV genomes that validate the divergence times of primate HPVs within niche-specific clades. Our analyses of the evolutionary dynamics of primate PVs, including specific focus on HPV16 variants, provide novel insights into the complex phylodynamic interactions between viruses and hosts and their pathologic outcomes.

## Results

### Genomic characterization of novel non-human primate papillomaviruses

**Fig 1:**
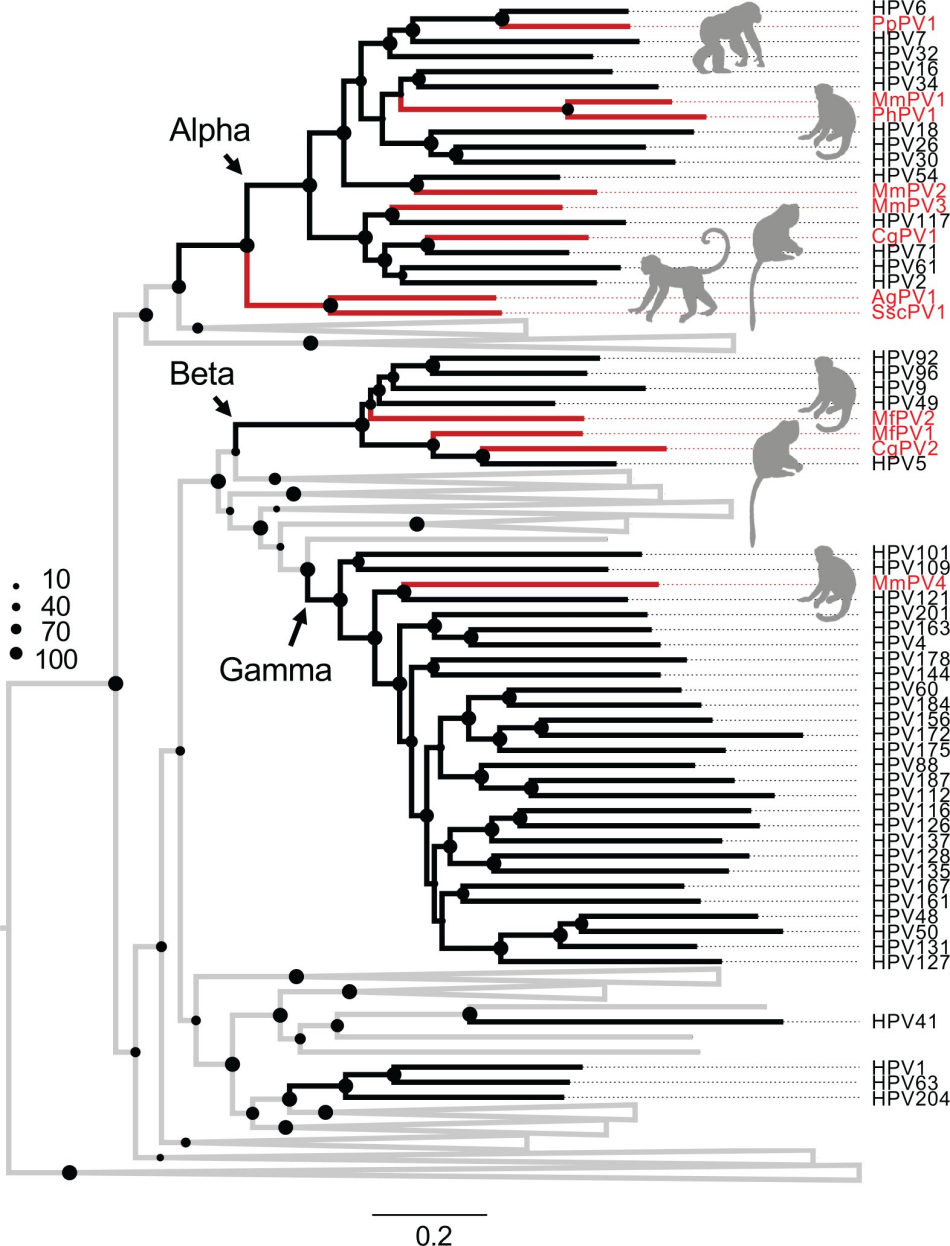
Phylogeny of primate papillomaviruses. A maximum likelihood phylogenetic tree was inferred from the concatenated nucleotide sequence alignment of 4 open reading frames (E1-E2-L1-L2) of 141 papillomavirus types representing 132 species (see PV list with hosts in S2 Table). The majority of analyzed primate papillomaviruses cluster into three distinct clades, Alpha-, Beta- and Gamma-PV genera, corresponding predominately to the anatomical sites (e.g., mucosal *vs*. cutaneous epithelium) where the viruses were originally isolated, rather than to the distinct host species. The branches represented by non-human primate papillomaviruses are highlighted in red. Non-primate papillomaviruses are collapsed and joined by grey lines (see comprehensive tree in S1 Fig). The dot sizes are proportional to the bootstrap percentage supports from RAxML.

In an effort to study the diversity of non-human primate PVs (NHP-PVs) to better understand the evolution of oncogenic HPVs, we screened cervicovaginal specimens from 10 adult female squirrel monkeys (*Saimiri sciureus*), and the paired oral, perianal, and genital samples from 8 adult rhesus monkeys (*Macaca mulatta*) (4 females and 4 males). Three novel *Saimiri sciureus* PV types (SscPV1, 2 and 3) and three novel *Macaca mulatta* PV types (MmPV2, 3 and 4) were isolated and characterized and had genomes ranging in size from 7424 bp to 8051 bp (S1 Table). All genomes contained five early genes (E6, E7, E1, E2, and E4), two late genes (L2 and L1), and an upstream regulatory region (URR) between L1 and E6 genes. Phylogenetic trees based on the nucleotide sequence alignment of the concatenated four open reading frames (ORFs) (E1, E2, L2, and L1) (Fig 1 and S1 Fig) or individual genes, e. g., E1 or L1 ORFs (S2 Fig, S3 Fig and S4 Fig) support a monophyletic clade grouping SscPV1/2/3 and howler monkey *Alouatta guariba* PV 1 (AgPV1, KP861980) [24] within the genus *Dyoomikronpapillomavirus*. MmPV2 and MmPV3 cluster into the genus *Alphapapillomavirus*, with the closest HPVs being HPV54 (within the species Alpha-13) and HPV117 (within the species Alpha-2), respectively. MmPV4 shares <70% of L1 ORF similarity with members of the species Gamma-10 (e.g., HPV121 and HPV130) and may represent a novel species within the genus *Gammapapillomavirus*.

### Intrahost divergence of primate papillomaviruses

We focused on HPV16 because it is the most prevalent and potent carcinogen among the oncogenic HPVs [5]. To interrogate HPV16 evolution using a molecular clock, we utilized HPVs and NHP-PVs characterized in our labs and by others where the host species separation times have been well established [25, 26]. This step is essential in order to validate a vertical mutation rate model suitable for HPV variants. This model estimates the mutation rate for infectious PVs over long periods of time and might differ from horizontal mutation rates not measured in this study.

Papillomaviruses have been identified in a wide range of NHP species, including Old World monkeys and apes (e.g., macaque, chimpanzee) and New World monkeys (e.g., squirrel monkey, brown howler) [24, 27–33]. Using a maximal likelihood algorithm and a nucleotide sequence alignment of the concatenated E1-E2-L2-L1 ORFs for 141 PV types representing each species or unique host (S2 Table), we found that the majority of primate PVs phylogenetically clustered into *Alphapapillomavirus*, *Betapapillomavirus*, or *Gammapapillomavirus* genera, corresponding predominantly to the anatomical sites where the viruses were originally isolated (e.g., mucosal or cutaneous epithelium), which was independent of the host species (Fig 1, S1 Fig and S2 Table). For example, MmPV1 is a rhesus macaque PV type (within the species Alpha-12) isolated from cervicovaginal cells that shares a most recent common ancestor (MRCA) with oncogenic mucosal HPV16 (within the species Alpha-9) but is distantly related to MmPV4 (within the genus *Gammapapillomavirus*), which was also isolated from a rhesus macaque. Since topological incongruence has been noted in the phylogenies of HPVs when trees are constructed with either late or early regions of the viral genomes [22, 34], we also examined the topologies of such trees. Although there was some incongruence, the majority of the primate PVs maintained their topological positions (see S2 Fig, S3 Fig and S4 Fig).

**Fig 2:**
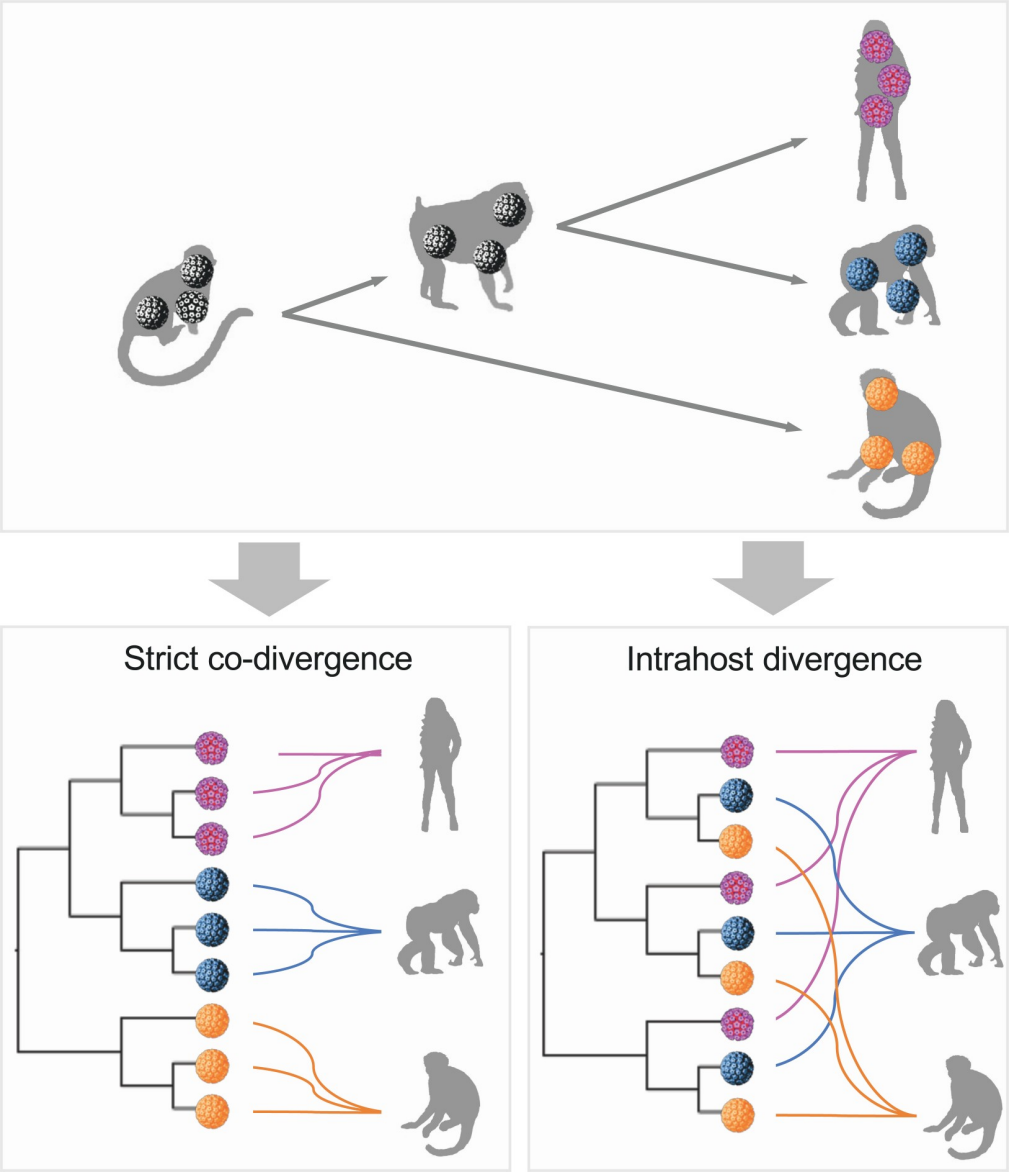
Schematic model of virus-host codivergence. Strict virus-host codivergence requires the evolutionary history of the pathogen to mirror that of its hosts. Clustering of viruses according to the host from which they were isolated should be observed. In addition, the divergence times of hosts and parasites should be similar (different colors highlight viruses infecting different primate host ancestors). Intrahost divergence can be defined according to specific phylogenetic criteria, such as niche-adaptation prior to coevolution in primate papillomaviruses, as opposed to clustering by hosts.

Fahrenholz’s proposal for strict codivergence of host and parasites states that the “parasite phylogeny mirrors that of its host,” indicating that specific pathogens isolated from an individual host species should be monophyletic to the exclusion of viruses from other host species (reviewed in de Vienne et al.) [35] (Fig 2). In the case of primate PVs, however, viruses infecting a given host species do not always cluster together, implying an ancient viral divergence model in which viral ancestors may have first split into separated viral clades corresponding to niche adaptation to specific host ecosystems (i.e., tissue tropism). Following host ancestor speciation, distinct but homophyletic viruses were transmitted to similar ecosystems (e.g., mucosal or cutaneous sites) between closely related host animals, resulting in the radiation observed in the extant primate PV tree where viruses sort by tissue tropism and not host species. This prediction was evaluated with a permutational multivariate analysis of variance (PERMANOVA) test [36] using primate PV nucleotide sequence pairwise distances, which revealed that tissue tropism (here defined by different genera) contributed to more of the variability of viral divergence (accounting for 26% of the total variance, p<0.001) than that of the host (6%, p<0.001) (Table 1).

**Table 1:** Permutational multivariate analysis of variance using primate papillomavirus pairwise distance.

4ORF nt	Df	Sums of Squares	Mean of Squars	F Model	R^2^	Pr (>F)
Host ^1^	1	0.3532	0.3532	4.5744	0.0606	<0.001
Genus ^2^	4	1.5396	0.3849	4.9854	0.2641	<0.001
Residuals	51	3.9375	0.0772		0.6754	

^1^ Host was grouped as "Human" and "Non-human primates (NHP)".

^2^ Genus was grouped as "*Alpha_Human*", "*Alpha_NHP*", "*Beta_Human*", "*Beta_NHP*", "*Gamma_Human*" and "*Gamma_NHP*".

### Co-divergence between human and non-human primate papillomaviruses

**Fig 3:**
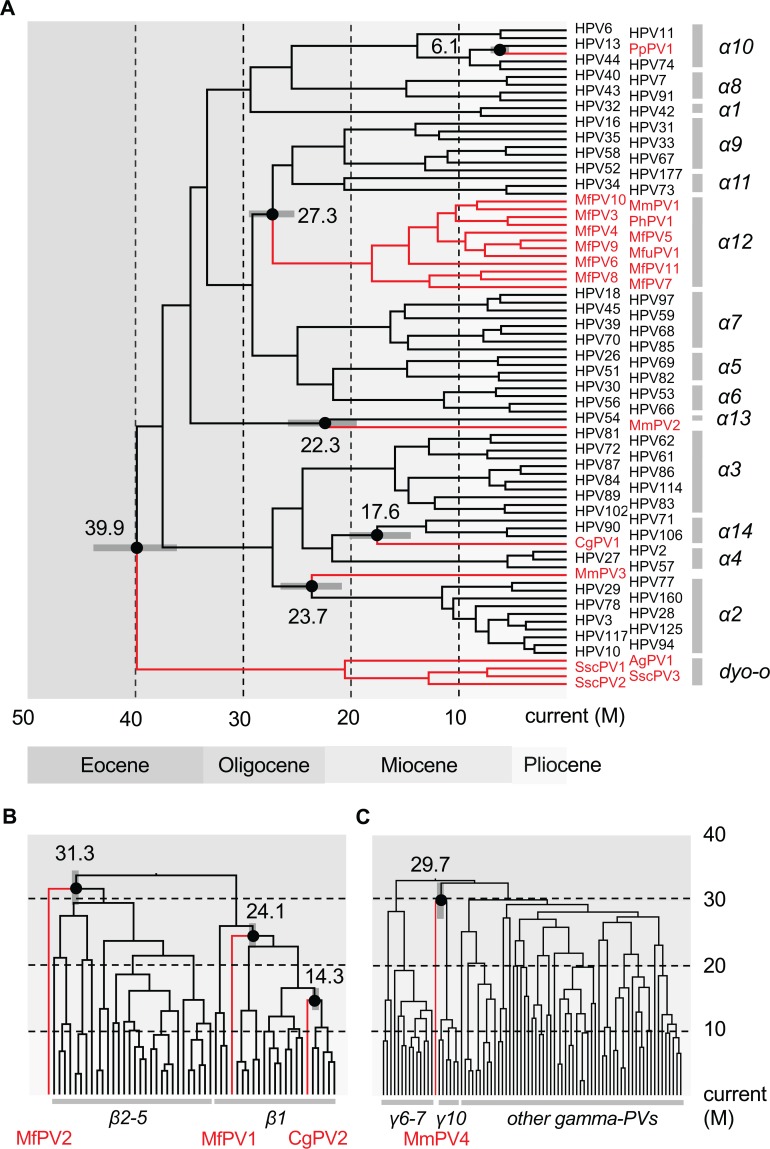
Divergence time estimation of primate papillomaviruses to their most recent common ancestors (MRCAs). A Bayesian MCMC method was used to estimate divergence times as described in the methods. Times were calculated separately for each genus, Alpha- **(A)**, Beta- **(B)** and Gamma-PVs **(C)**. Branch lengths are proportional to divergence times. The branches in red refer to non-human primate papillomaviruses. Numbers above the nodes with circles are the mean estimated divergence time in million years (M) between human and non-human papillomavirus clades. The bars in grey represent the 95% highest posterior density (HPD) interval for the divergence times (see details in S5 Fig, S6 Fig and S7 Fig, respectively). Panels B and C show time on the Y-axis and phylogeny on the X axis.

To estimate the divergence times of primate PVs from their MRCAs, we used a Bayesian statistical framework employing previously established PV evolution rates [17]. Infectious PVs have been shown to have a slow mutation rate based on the observations that these double-stranded DNA viruses use the host cell DNA replication machinery, characterized by high fidelity, proofreading capacity, and post-replication repair mechanisms [37]. Since primate PVs, taken together, do not follow strict viral-host codivergence, each genus was evaluated separately to estimate divergence times. A combination of relaxed lognormal molecular clock and coalescent constant population models provided the best performance using the phylogenetic tree as shown in Fig 3A. The *Alphapapillomavirus*–*Dyoomikronpapillomavirus* split from a MRCA around 39.9 million years ago (mya) (95% highest posterior density (HPD), 36.4–43.7 mya) (Fig 3A, S2 Fig and Table 2) is consistent with the time frame of the split between New World and Old World primate ancestors [26].

**Table 2:** Divergence time estimation of Alphapapillomavirus and Dyoomikronpapillomavirus types.

Calibration	Clock Model	Tree Prior	AICM value	AICM difference	Log marginal likelihood	MRCA (million years ago, mya)											
						Alpha vs Dyoomega (mean)	95% HPD Interval	α9/11 vs α12 (mean)	95% HPD Interval	HPV54 vs MmPV2 (mean)	95% HPD Interval	α2 vs MmPV3 (mean)	95% HPD Interval	α14 vs CgPV1 (mean)	95% HPD Interval	HPV13 vs PpPV1 (mean)	95% HPD Interval
3 Cali.	Relaxed	Bayesian	719437	47	-359153.05	39.7	[36.2, 43.5]	27.2	[25.2, 29.3]	22.5	[19.0, 26.1]	23.8	[21.2, 26.4]	17.7	[15.7, 19.8]	6.1	[5.4, 6.8]
**3 Cali.**^**#**^	**Relaxed**	**Constant**	**719391**	**-**	**-359153.10**	**39.9**	**[36.4, 43.7]**	**27.3**	**[25.3, 29.4]**	**22.3**	**[19.1, 25.7]**	**23.7**	**[21.4, 26.1]**	**17.6**	**[15.6, 19.8]**	**6.1**	**[5.5, 6.8]**
3 Cali.	Relaxed	Yule	719399	8	-359153.30	39.6	[36.1, 43.2]	27.2	[25.1, 29.2]	22.2	[19.0, 25.6]	23.6	[21.3, 26.1]	17.6	[15.7, 19.7]	6.2	[5.5, 6.8]
3 Cali.	Strict	Bayesian	721072	1681	-360478.67	35.6	[32.7, 38.3]	25.9	[23.9, 27.8]	17.8	[16.3, 19.5]	21.1	[19.3, 23.0]	15.6	[14.3, 17.0]	5.3	[4.8, 5.7]
3 Cali.	Strict	Constant	721074	1684	-360478.92	35.7	[32.9, 38.5]	25.9	[24.0, 27.9]	17.9	[16.3, 19.5]	21.1	[19.4, 23.0]	15.6	[14.3, 17.0]	5.2	[4.8, 5.7]
3 Cali.	Strict	Yule	721073	1682	-360478.78	35.5	[32.9, 38.5]	25.8	[24.0, 27.8]	17.8	[16.2, 19.4]	21.1	[19.4, 22.9]	15.5	[14.3, 16.9]	5.2	[4.8, 5.7]
No	Relaxed	Bayesian	719633	242	-359275.52	33.3	[30.4, 36.1]	25.0	[23.4, 26.6]	16.9	[14.7, 19.3]	18.9	[17.5, 20.4]	13.8	[12.6, 15.1]	4.7	[4.1, 5.4]
No	Relaxed	Constant	719681	290	-359270.59	34.2	[31.6, 37.1]	24.2	[22.7, 25.7]	15.4	[13.5, 17.3]	21.0	[19.1, 22.7]	15.0	[13.5, 16.5]	4.5	[3.8, 5.2]
No	Relaxed	Yule	719636	246	-359274.64	33.2	[30.5, 36.3]	25.1	[23.5, 26.8]	17.0	[14.9, 19.2]	18.9	[17.5, 20.5]	13.9	[12.5, 15.2]	4.6	[3.9, 5.4]
No	Strict	Bayesian	721038	1647	-360471.10	31.0	[29.6, 32.3]	22.5	[21.7, 23.4]	15.6	[14.7, 16.6]	18.5	[17.6, 19.5]	13.6	[12.9, 14.3]	4.4	[4.1, 4.8]
No	Strict	Constant	721037	1646	-360471.21	31.0	[29.7, 32.4]	22.6	[21.7, 23.4]	15.6	[14.7, 16.6]	18.5	[17.5, 19.4]	13.6	[12.9, 14.3]	4.4	[4.1, 4.7]
No	Strict	Yule	721039	1648	-360471.24	31.0	[29.6, 32.3]	22.6	[21.7, 23.5]	15.6	[14.6, 16.6]	18.5	[17.5, 19.4]	13.6	[12.9, 14.3]	4.4	[4.1, 4.7]

^#^ The best models determined by the AICM test are highlighted in bold.

Similar virus-host codivergence events were observed between Old World monkey PVs and their closest HPV relatives, and were estimated to approximately 14–31 mya (Fig 3, S5 Fig, S6 Fig and S7 Fig). For example, the species Alpha-12 (PVs mainly isolated from genital lesions of macaques) split from a MRCA with the species Alpha-9 (represented by oncogenic genital HPV16) around 27 mya coincided with the time span of the speciation between macaques and apes/humans that occurred approximately 25 mya [38, 39]. An enigmatic observation in these data is the clustering of macaque PVs (e.g., MfPV3) and baboon PV (*Papio hamadryas* PV 1, PhPV1) within the species Alpha-12 group, suggesting either a recent viral transmission between macaque and baboon monkeys, or a more complex phylogeny of the sub-family *Cercopithecinae*. The majority of distinct human PV types arose during the end of the Miocene and/or the beginning of the Pliocene epoch coincident with the divergence of humans and chimpanzees occurring around 6–8 mya (Fig 3) [40].

**Fig 4:**
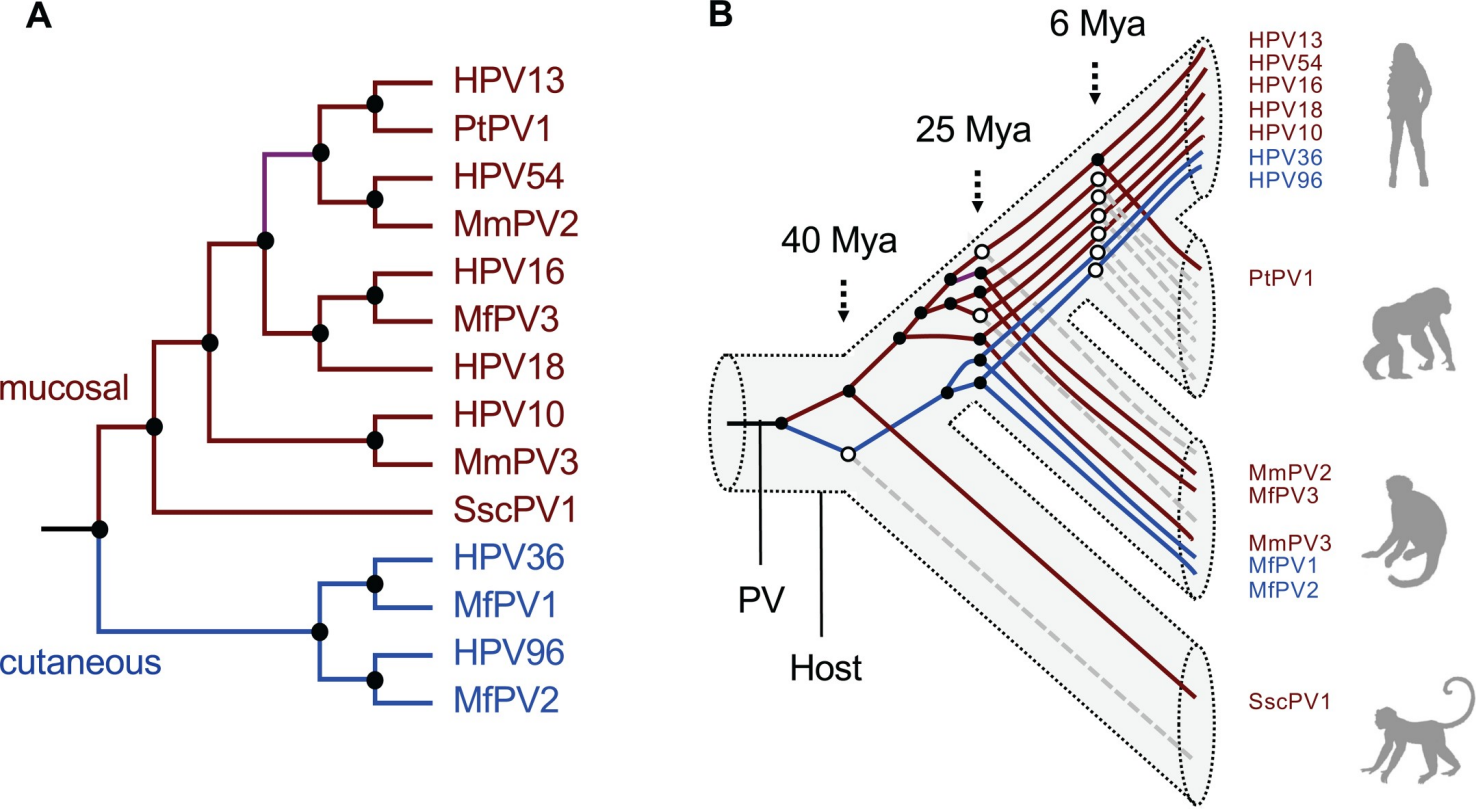
Schematic model of virus-host codivergence of primate papillomaviruses. **(A)** A schematic topology of representative primate papillomaviruses. The branch colors represent viruses with specific host niche adaptation (brown–isolated from mucosal tissues, blue–isolated from cutaneous tissue). **(B)** Model of phylogeny and divergence of primate papillomaviruses. In this model, one or more primate papillomavirus ancestors evolved to colonize distinct host ecosystems prior to the speciation of a primate ancestor. A process of further viral adaptation to colonize more specific host ecosystems (represented by black circles at the nodes) may have followed upon host speciation, resulting in the radiation observed in the extant primate papillomavirus tree. The broken lines in grey (starting from open circles) represent clades for which specific HPV species lack detectable non-human primate counterparts. The 3-dimentional structure represent host phylogeny.

The divergence times and tree topologies support a model of intrahost divergence of primate PVs in which ancient viruses diverged and adapted to specific host ecosystems (e.g., tissue tropism or different types of epithelial cells) within an ancestral host animal lineage (e.g., the MRCA of primate animals) (Fig 4). Following periods of host speciation, continuing intrahost viral divergence events occurred as distinct but phylogenetically related viral types were transmitted to similar host ecosystems by the closely related host animals. This pattern of ancient viral divergence coupled to niche adaptation may explain, for example, the differences in the prevalence of HPV16 and HPV18 between squamous cell carcinomas and adenocarcinomas of the cervix [41]. This difference might represent the emergence of further viral adaptation to different ecological niches within the cervix, one dominated by stratified squamous epithelium the other by columnar epithelium, respectively [42]. The fact that we do not observe similar or parallel diversity of NHP-PVs compared to HPVs (broken lines in right panel of Fig 4B) could be due, in part, to reduced sampling effort, limited population size of NHPs, bottlenecks of viral transmission, and/or restricted host migration.

### Molecular evolution and geographic distribution of HPV16 variants

Next, we constructed a phylogenetic tree of HPV16 variants based on 212 complete genomes to classify variant lineages and sublineages (S3 Table). The tree topology shows two deeply separated clades corresponding to the previously classified Eurasian and African lineages (S8 Fig), with a mean nucleotide sequence difference of 1.72% ± 0.09% (S4 Table). The African lineage variants were more than twice as diverse (intragroup mean difference of 0.77% ± 0.04%) as the Eurasian variants (0.32% ± 0.02%). Since geographic nomenclature systems suffer from sampling biases and preconceived notions about virus ancestry, we utilized an agnostic alphanumeric nomenclature based on HPV16 phylogeny and complete genome nucleotide differences to assign HPV16 variants into four lineages designated A, B, C, and D. Each lineage could be divided into four sublineages (A1-4, B1-4, C1-4, and D1-4), based on previously described criteria (S9 Fig) [43]. The previously named Asian (As) and North American 1 (NA1) variants are designated sublineages A4 and D1, respectively [44]. The maximum pairwise difference between the most diverse isolates, from sublineages A1 and D3, was 2.23%.

**Fig 5:**
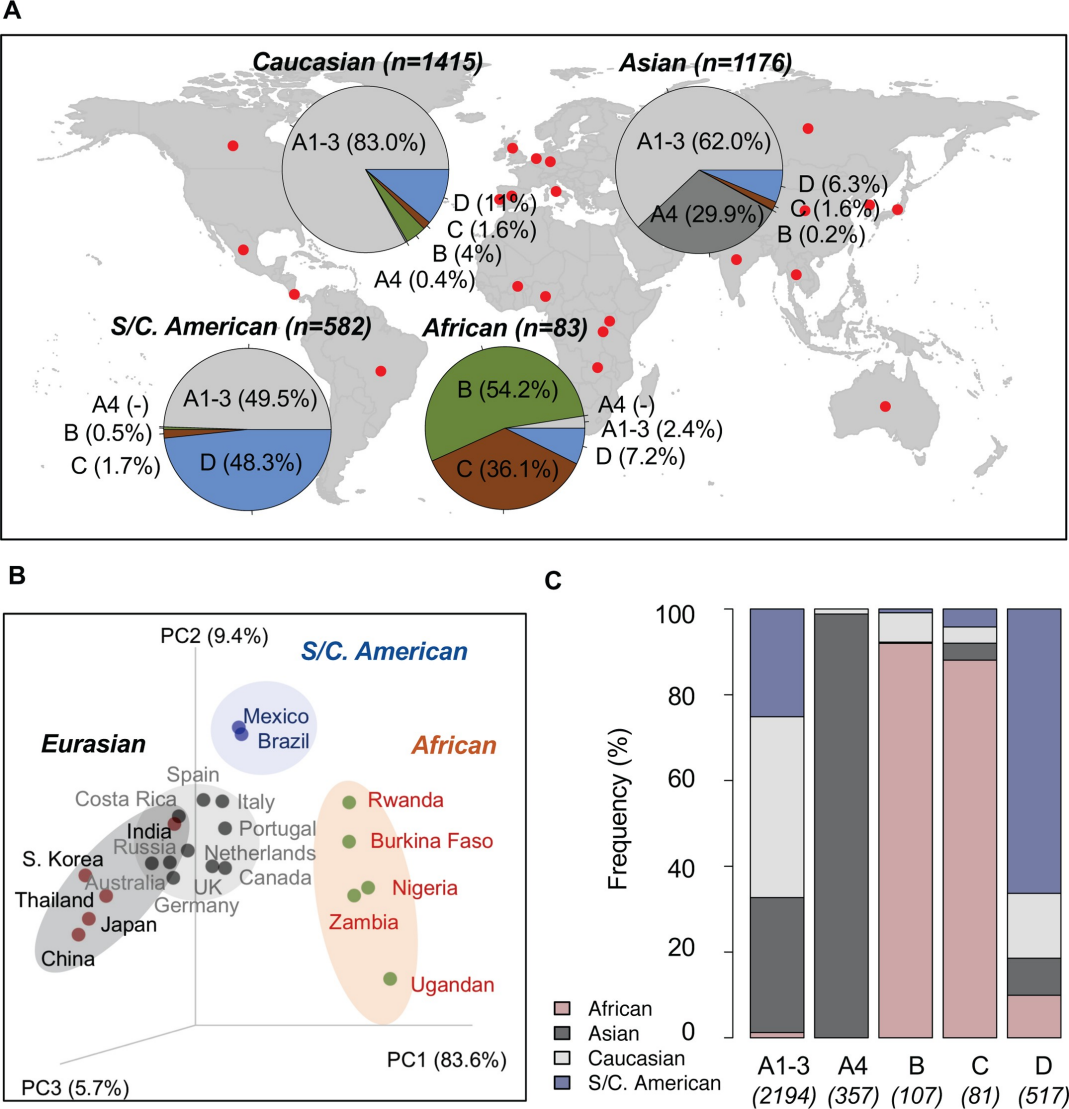
Geographic distribution of HPV16 variants. **(A)** A total of 3256 HPV16 variants with known geographic origin from 22 countries/regions (see details in Table 3) were assigned into lineage/sublineage and summarized by geographic group in the pie charts. **(B)** Principle component analysis using a weighted UniFrac algorithm clustered different study cohorts into three distinct groups, namely African, Eurasian (Asian and Caucasian) and South/Central American, mainly associated with a predominant population from which viruses were sampled. **(C)** Relative frequency of HPV16 lineages/sublineages distribute into four major geographic populations (African, Asian, Caucasian, and South/Central American).

Based on single-nucleotide polymorphism (SNP) patterns and phylogenetic tree topologies, we assigned 3256 HPV16 partial sequences from 22 countries/studies into variant lineages and sublineages using maximum likelihood methods (Table 3). As shown in the summarized charts of HPV16 phylogeography (Fig 5A), isolates from Asians and Caucasians (Australians/Europeans, and North Americans) were predominantly represented by A variants, with abundances of 92% and 83%, respectively. The majority of A4 variants (352/357, 99%) were from Asian individuals. Within the African population, 90% of HPV16 infections were B and C lineages. HPV16 variants in South/Central Americans were equally assigned as A1-3 (50%) and D (48%). Using a weighted UniFrac algorithm, variants were well clustered into groups (African, Eurasian, and South/Central American) corresponding to the geographic origin of the isolates (Fig 5B). Globally, A1-3 sublineages were the most widespread; whereas, the D lineages were detectable at low prevalences in many populations outside of South/Central Americans, such as in Caucasian (11%), African (7%), and Asian (6%) individuals (Fig 5C). In contrast, A4 and B/C lineages were rarely found outside of Asian and African populations, respectively.

**Table 3:** HPV16 variant assignment with known geographic origin.

Country	Geographic/ethnic origin	Total	A1-3	A4	B	C	D	References
Burkina Faso	African	16	0	0	4	11	1	[100]
Nigeria	African	21	0	0	12	9	0	[101]
Rwanda	African	20	0	0	10	6	4	[102]
Ugandan	African	5	0	0	5	0	0	[103]
Zambia	African	21	2	0	14	4	1	[104]
China	Asian	361	148	197	2	11	3	[105–107]
India	Asian	488	427	7	0	0	54	[108, 109]
Japan	Asian	57	31	25	0	0	1	[110]
Korea	Asian	56	9	38	0	1	8	[111]
Thailand	Asian	214	114	85	0	7	8	[112, 113]
Australia	Caucasian	34	28	4	0	0	2	[132]
Canada	Caucasian	32	25	0	5	1	1	GenBank^#^
Costa Rica	Caucasian	693	604	1	1	2	85	[9]
Germany	Caucasian	24	24	0	0	0	0	[114]
Italy	Caucasian	197	143	0	18	4	32	[115–117]
Netherlands	Caucasian	67	59	0	3	1	4	[119, 120]
Portugal	Caucasian	187	139	0	22	8	18	[121]
Russia	Caucasian	50	48	0	0	0	2	[122]
Spain	Caucasian	75	59	0	3	1	12	[123]
United Kingdom	Caucasian	56	46	0	5	5	0	[124]
Brazil	South/Central American	200	98	0	3	10	89	[125–127]
Mexico	South/Central American	382	190	0	0	0	192	[128–131]
Total	-	3256	2194	357	107	81	517	

^#^ GenBank/NCBI accession numbers of GQ465877 to GQ465902, and GQ479006 to GQ479011.

### Divergence time estimation of HPV16 variants and other oncogenic HPV type variants

**Fig 6:**
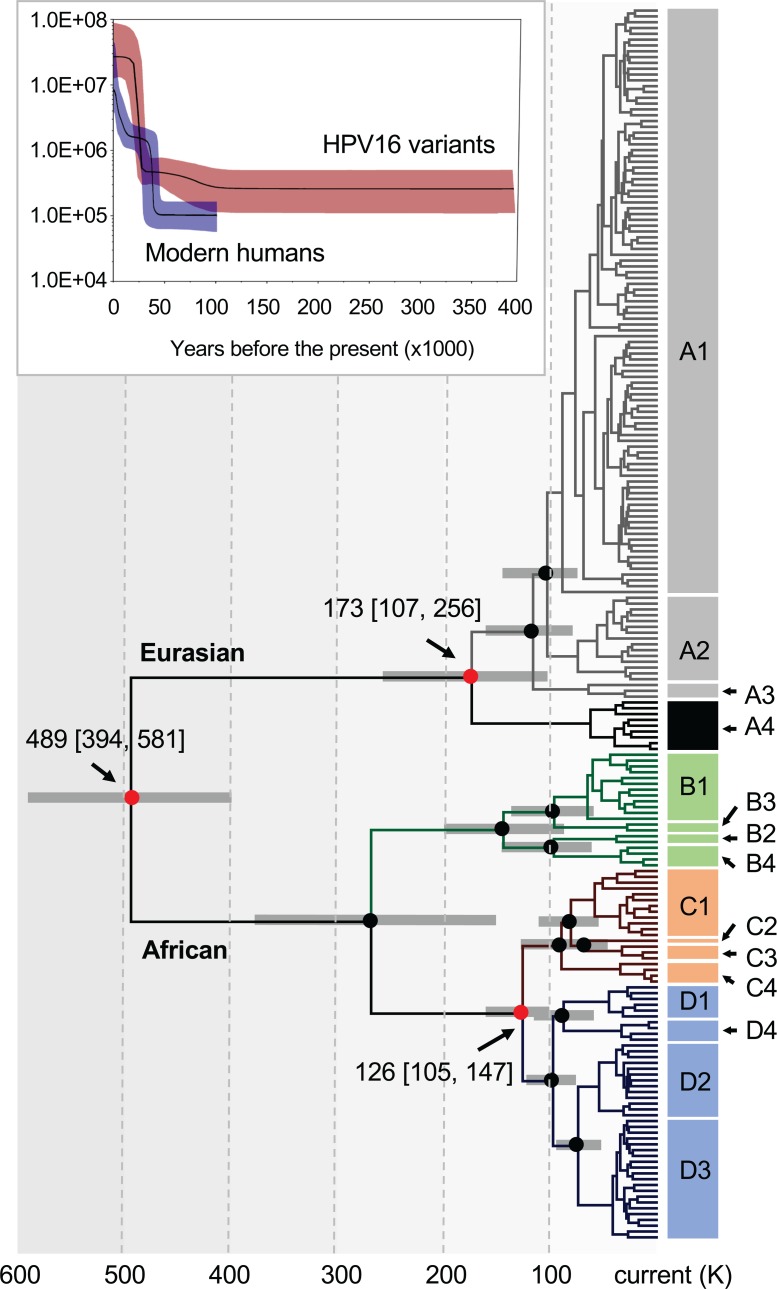
Divergence time estimation of HPV16 complete genome variants. A Bayesian MCMC method was used to calculate the divergence times of HPV16 complete genome variants from their most recent common ancestors, as described in the methods. The nodes highlighted with red circles indicate divergence times of the split between HPV16 A and non-A lineages, between A1-3 and A4 sublineages, and between C and D lineages. Branch lengths are proportional to divergence times scaled in thousands of years (K). Grey bars indicate the 95% highest posterior density (HPD) for the corresponding divergence age (see details in S10 Fig). Colors in branches represent distinct HPV16 variant lineages/sublineages. The plot on top of the tree is a Bayesian skyline estimation based on 311 present-day human mtDNA sequences (without the loop region) from geographically diverse populations and 212 HPV16 complete genome variants with a similar geographical distribution. The median posterior estimates (the product of the effective population size *N*_*e*_ and the generation length *g* in years) throughout the given time period are illustrated with lines in black. The dark blue (humans) and dark red (HPV16) areas give the 95% HPD interval of these estimates.

The molecular clock models used to estimate the divergence times of primate PVs support a scenario of virus-host codivergence after the virus has adapted to a specific host ecosystem. Using a similar Bayesian Markov chain Monte Carlo (MCMC) framework, we initially applied six combinations of clock models to estimate the divergence of HPV16 variants from their MRCA, without any prior assumption of virus-host codivergence (Table 4, no calibration). Interestingly, a combination of the relaxed lognormal molecular clock and coalescent Bayesian skyline models indicated that HPV16 A and BCD had divided around 618.5 thousand years ago (kya) (95% HPD: 331.5–996.1). This estimation is within the time span of the separation between *Homo sapiens* and archaic hominins (e.g., Neanderthal/Denisova) but around two-five times longer than the estimated modern *Homo sapiens* divergence time (ca. 150–200 kya) [45] indicative of an ancient divergence of HPV16 variants prior to the emergence of modern human ancestors. Based on the geographic distribution of HPV16 variants above, we then used an archaic *hominin-host-switch* (*HHS*) scenario to calibrate the divergence time between HPV16 A and non-A variants (500 kya, 95% HPD: 400–600), and a *modern-out-of-Africa* (*MOA*) scenario between BC and D variants (90 kya, 95% HPD: 60–120). When time calibrations were introduced into the phylogenetic tree, the *HHS* scenario showed the strongest support for time inference and estimated an initial divergence of HPV16 variants at approximately 489 kya (95% HPD: 394–581), predating the *out-of-Africa* migration of modern humans (ca. 60–120 kya) (Fig 6 and S10 Fig) [46, 47]. In addition, the demographic model of the Bayesian skyline plot for the population function through time showed a recent exponential expansion of the effective population size of present-day HPV16 occurring in the last 25 kya, lagging behind the growth of modern human populations (starting from the last 40–50 kya) (see the top panel of Fig 6). This plot most likely reflects the concurring increase and mobility of modern human populations and present-day virus populations in the last epoch.

**Table 4:** Divergence time estimation of HPV16 variant lineages.

Calibration	Clock Model	Tree Prior	AICM value	AICM difference	Log marginal likelihood	MRCA (thousand years ago, kya)							
						A vs BCD (mean)	95% HPD Interval	A1/3 vs A4 (mean)	95% HPD Interval	B vs C/D (mean)	95% HPD Interval	C vs D (mean)	95% HPD Interval
No	Relaxed	Bayesian	47641	2	-23609.43	618.5	[331.5, 996.1]	199.9	[122.8, 306.4]	401.0	[250.1, 584.0]	260.0	[161.9, 381.3]
No	Relaxed	Constant	47644	4	-23606.09	1025.0	[450.4, 1843.4]	360.3	[172.0, 600.3]	667.8	[302.9, 1140.2]	439.3	[217.5, 715.0]
No	Relaxed	Yule	47655	16	-23613.10	337.2	[226.0, 460.0]	187.4	[127.1, 259.5]	286.9	[195.9, 385.3]	219.8	[141.2, 305.3]
No	Strict	Bayesian	47886	247	-23784.30	519.3	[387.8, 663.1]	177.1	[126.8, 235.1]	335.0	[246.6, 432.5]	213.1	[157.7, 277.8]
No	Strict	Constant	47852	213	-23777.28	539.8	[409.0, 686.4]	195.4	[139.4, 255.3]	353.1	[263.9, 456.3]	227.1	[168.8, 293.3]
No	Strict	Yule	47829	190	-23772.25	472.5	[355.6, 604.0]	176.4	[128.6, 230.9]	312.5	[235.2, 405.2]	204.0	[153.5, 264.3]
**2 Cali.**^**#**^	**Relaxed**	**Bayesian**	**47639**	**-**	**-23609.83**	**488.9**	**[394.2, 581.0]**	**172.7**	**[106.6, 255.8]**	**266.1**	**[164.1, 386.8]**	**125.5**	**[104.8, 147.2]**
2 Cali.	Relaxed	Constant	47651	11	-23610.23	513.1	[421.0, 601.5]	259.7	[153.3, 383.0]	341.9	[220.2, 496.2]	132.8	[111.1, 154.2]
2 Cali.	Relaxed	Yule	47671	32	-23613.98	403.7	[316.1, 493.8]	183.0	[128.0, 253.1]	234.1	[166.8, 314.9]	130.9	[110.8, 151.5]
2 Cali.	Strict	Bayesian	47900	261	-23787.00	440.1	[384.4, 495.1]	150.9	[119.8, 184.4]	255.5	[213.7, 296.9]	142.6	[125.1, 160.7]
2 Cali.	Strict	Constant	47854	215	-23779.58	449.2	[397.0, 506.4]	165.0	[133.9, 198.3]	264.7	[222.4, 305.0]	147.6	[130.8, 165.8]
2 Cali.	Strict	Yule	47824	185	-23774.85	413.6	[362.4, 469.7]	153.3	[124.5, 184.8]	246.4	[208.3, 286.7]	143.1	[126.5, 160.4]

^#^ The best models determined by the AICM test are highlighted in bold.

**Fig 7:**
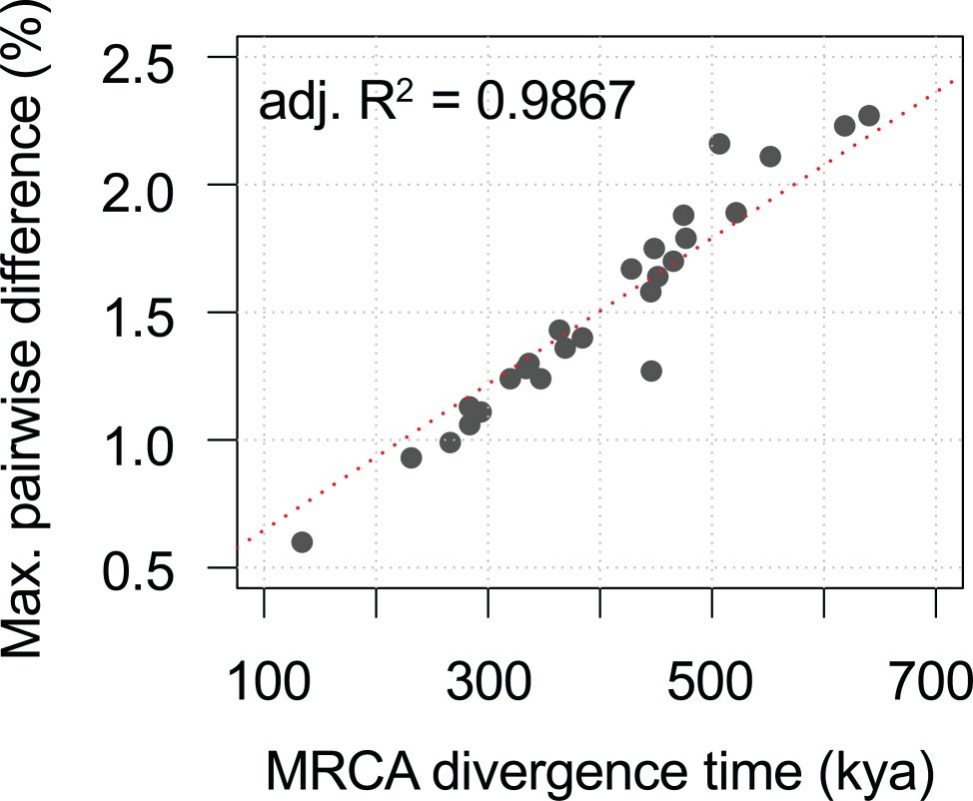
Ancient HPV variant codivergence with archaic hominins. The plot shows the correlation between divergence time (X-axis) of HPV variants from the most recent common ancestors and genomic diversity (Y-axis) of HPV variants. The Alpha-3 (HPV61), Alpha-5 (HPV26, 51, 69, 82), Alpha-6 (HPV30, 53, 56, 66), Alpha-7 (HPV18, 39, 45, 59, 68, 70, 85, 97), Alpha-9 (HPV16, 31, 33, 35, 52, 58, 67), Alpha-10 (HPV6, 11), Alpha-11 (HPV34, 73), and Alpha-13 (HPV54) variants from previous publications were included. The adjusted R^2^ value indicating the correlation between sequence diversity and divergence time of HPV type variants was calculated using the linear model (*lm*) function in R.

**Fig 8:**
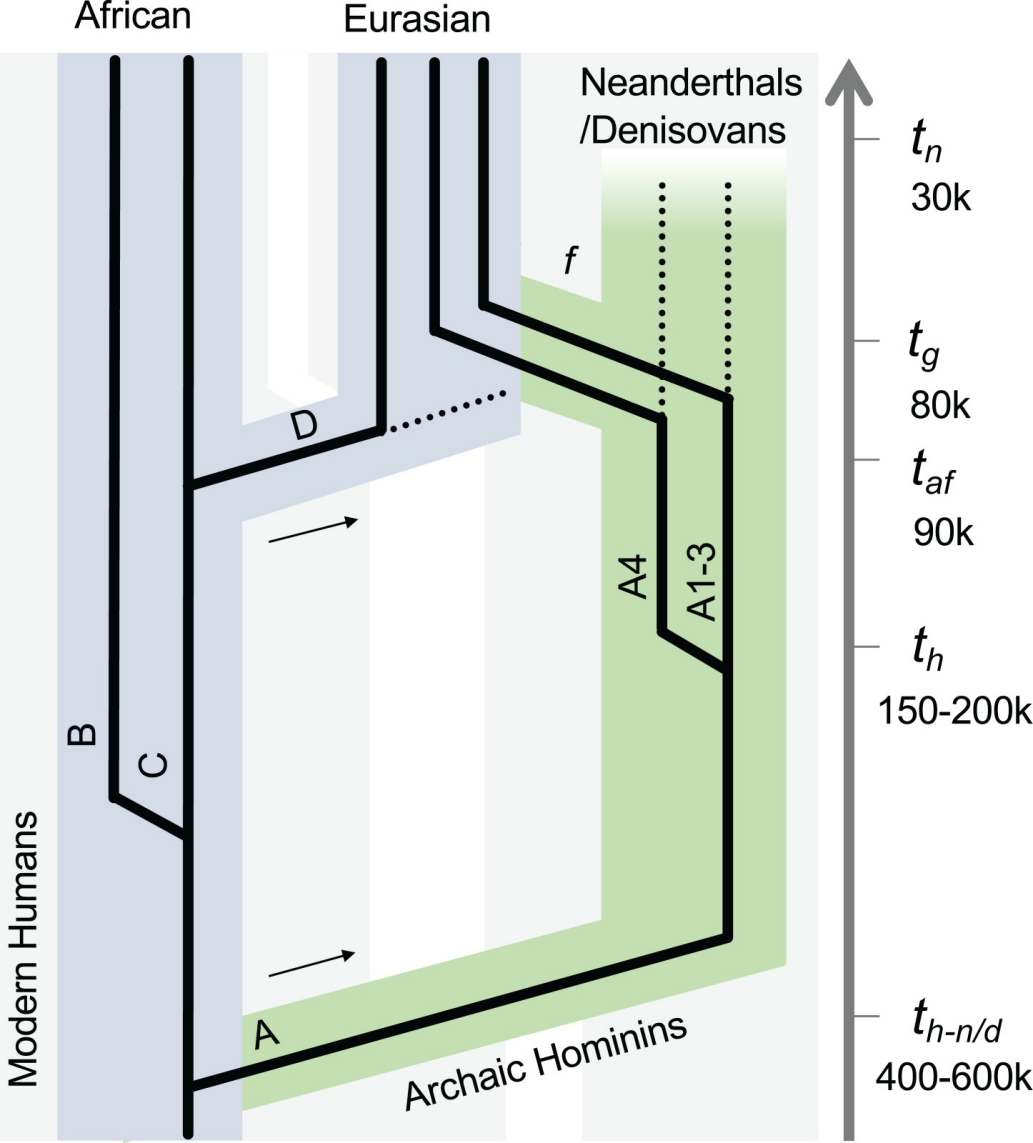
Schematic illustration of HPV16 codivergence with archaic hominins. The model was based on HPV16 variant divergence time estimation, phylogenetic topology, and geographic distribution that superimposes an ancestral viral transmission between Neanderthals/Denisovans and modern human populations. The early divergence event among deeply separated HPV16 variant lineages (A *vs*. BCD) suggests ancient virus-host codivergence following the speciation of modern humans and archaic hominins (e.g., Neanderthals and Denisovans) from their most recent common ancestors. The gene flow through host interbreeding between archaic hominins allowed viral transmission from Neanderthals/Denisovans to modern humans. *t*_*h-n/d*_ denotes the splitting time between Neanderthals/Denisovans and modern humans, *t*_*h*_ represents the speciation of modern humans. *t*_*af*_ indicates the era of population expansion of modern humans walking out-of-Africa. *t*_*g*_ indicates the time of gene flow (f) that may have occurred between modern humans and Neanderthals/Denisovans. *t*_*n*_ estimates the extinction of Neanderthals/Denisovans. The arrows indicate the out-of-Africa migration events of archaic and modern human populations. The broken lines indicate potential extinction of viral variants. Branch lengths and widths are not drawn to scale.

We observed a similar divergence timeframe for other HPV variants, splitting from their MRCAs approximately 300–600 kya and showing a strong correlation between evolution times and genomic diversities (Fig 7, Table 5). In all cases, the deep separation between HPV16 variant lineages A and BCD (and the deepest lineage separations of other HPV variants) suggests an ancient virus-host codivergence, coinciding with the split between archaic Neanderthal/Denisova and modern human ancestors from their MRCA (Fig 8). Neanderthals spread out over Eurasia with at least two populations splitting approximately 77–114 kya from each other based on analysis of archaic genomes from Vindija, Mezmaiskaya (Caucasus), and Denisova (Siberia) [48]. This time period corresponds to the diversion of HPV16 A sublineages and in particular the split of A4 from A1/2/3 and the emergence of HPV16 A4 in Asia, likely representing independent transmission of A4 from archaic hominins to modern humans in the east.

**Table 5:** Estimation of divergence time (thousand years ago, kya) of HPV variants from the most recent common ancestor (MRCA).

HPV	Number	MRCA divergene (kya)		Estimated Rate (10x^-8^)		Maximum nuc. difference		Variant lineage/	Deepest
type	of isolates	Mean	95% HPD Interval	Mean	95% HPD Interval	Mean	s.d.	sublineage	separated clades
HPV61	10	640.2	[280.9, 1101.7]	1.77	[0.94, 2.60]	2.27%	0.17%	A1-2, B, C	A/B vs C
HPV16	212	618.5	[331.5, 996.1]	2.09	[1.85, 2.31]	2.23%	0.16%	A1-4, B1-4, C1-4, D1-4	A vs B/C/D
HPV18	46	552.1	[428.5, 704.6]	1.80	[1.61, 1.96]	2.11%	0.16%	A1-5, B1-3, C	A vs B/C
HPV30	16	521.5	[340.4, 717.7]	1.79	[1.43, 2.12]	1.89%	0.17%	A1-5, B	A vs B
HPV52	150	506.7	[203.7, 989.6]	1.84	[1.41, 2.26]	2.16%	0.17%	A1-2, B1-3, C1-2, D	A/B/C vs D
HPV53	27	476.7	[404.8, 549.7]	1.84	[1.73, 1.92]	1.79%	0.13%	A, B, C, D1-4	A/B vs C/D
HPV68CDEF	15	474.6	[356.6, 614.4]	1.86	[1.38, 2.34]	1.88%	0.12%	C1-2, D1-2, E, F1-2	C/F vs D/E
HPV58	138	465.4	[315.4, 659.8]	1.74	[1.53, 1.95]	1.70%	0.17%	A1-3, B1-2, C, D1-2	A vs B/C/D
HPV70	9	451.5	[336.7, 572.7]	1.82	[1.51, 2.09]	1.64%	0.12%	A, B	A vs B
HPV66	14	448.4	[257.7, 638.7]	1.83	[1.36, 2.30]	1.75%	0.14%	A, B1-2	A vs B
HPV54BC	5	445.8	[162.9, 845.7]	1.88	[0.84, 3.12]	1.27%	0.13%	B, C1-2	B vs C
HPV6	190	445.3	[366.0, 528.4]	1.84	[1.80, 1.88]	1.58%	0.13%	A, B1-5	A vs B
HPV45	33	428.0	[288.3, 577.8]	1.77	[1.47, 2.07]	1.67%	0.15%	A1-3, B1-3	A vs B
HPV11	78	384.2	[279.2, 483.3]	1.85	[1.67, 2.02]	1.40%	0.15%	A1-4, B	A vs B
HPV31	88	368.8	[230.9, 541.3]	1.76	[1.55, 1.96]	1.36%	0.14%	A1-2, B1-2, C1-4	A/B vs C
HPV73	16	363.6	[175.9, 596.6]	1.77	[1.08, 2.41]	1.43%	0.12%	A1-2, B	A vs B
HPV34AB	8	346.9	[270.2, 421.5]	1.84	[1.65, 2.03]	1.24%	0.11%	A1-2, B	A vs B
HPV59	8	336.5	[259.8, 413.6]	1.83	[1.61, 2.05]	1.30%	0.13%	A1-3, B	A vs B
HPV51	29	333.8	[242.9, 429.1]	1.82	[1.56, 2.04]	1.28%	0.12%	A1-4, B1-2	A vs B
HPV33	22	319.7	[211.3, 441.4]	1.81	[1.44, 2.13]	1.24%	0.12%	A1-2, B, C	A vs B/C
HPV39	20	293.6	[219.3, 374.2]	1.83	[1.61, 2.02]	1.11%	0.11%	A1-2, B	A vs B
HPV67	8	283.5	[219.2, 350.1]	1.83	[1.64, 2.00]	1.06%	0.11%	A1-2, B	A vs B
HPV82AB	11	283.3	[202.2, 369.3]	1.89	[1.37, 2.47]	1.13%	0.10%	A1-3, B1-2	A vs B
HPV56	12	266.1	[177.5, 354.9]	1.80	[1.44, 2.13]	0.99%	0.11%	A1-2, B	A vs B
HPV69	7	231.3	[173.9, 294.7]	1.81	[1.45, 2.07]	0.93%	0.10%	A1-4	A1/2 vs A3/4
HPV35	24	133.8	[98.0, 173.9]	1.81	[1.61, 1.99]	0.60%	0.07%	A1-2	A1 vs A2

Time was estimated using uncorrelated lognormal distribution molecular clock and coalescent Bayesian skyline models without calibration.

## Discussion

In this work, we used a Bayesian MCMC framework to estimate the divergence times of primate PVs and propose an early ancient intrahost viral divergence model (i.e., niche adaptation) followed by viral-host coevolution. This form of viral evolution has been documented for polyomaviruses [49], herpesviruses [50], and some retrovirus genera [51]. With the assumption of host niche adaptation as a fundamental process, the estimation of primate PV divergence times within niche-specific clades mirrors that of the primate host evolutionary history (Fig 4). It is clear that the evolutionary history of these well adapted, slowly evolving PVs may be significantly more complex than previously appreciated [37]. The implication of host niche adaptation of primate PVs preceding virus-host codivergence suggests a critical role for viral genetic heterogeneity and natural selection. The origin of viral genetic determinants of cervical niche adaptation further supports the hypothesis that a group of well-evolved viral genotypes also contain the determinants for cervical cancer, since this phenotype cannot exert selective pressure, as it does not support the production of infectious virus. It may also explain why a large set of cervicovaginal macaque PVs (within the species Alpha-12) associated with cervical neoplasia shares a common origin with the high-risk clade of human PVs (e.g., Alpha-9) (Fig 3A) [6, 27]. Our findings provide a framework for studying the past evolution of primate PVs infecting the genital tract niche and support a molecular clock based on phylogeny, since the generation time of PVs can only be extrapolated from empiric data based on coevolution models [17, 52].

We used this well-supported molecular clock model to estimate the divergence times of HPV16 variants. HPV16 is the most common oncogenic HPV type and shows diversity in persistence and carcinogenicity [53–55], suggesting further biological differences between variant lineages. We observed specific geographic/ethnic dispersals of HPV16 variants, such as A4 predominance in Asian populations and BC predominance in African populations. The estimated divergence times between HPV16 A and BCD variants largely predated that of the *out-of-Africa* migration of modern human populations, consistent with a previously reported archaic *hominin-host-switch* scenario [19, 20]. One interpretation of the data implies that the present-day Eurasian HPV16 A variants were probably the products of multiple interactions between Neanderthals/Denisovans and modern *Homo sapiens* established during sexual contact after a long period of separation (e.g., 400–600 kya). This notion of viral sexual transmission between groups is reflected in the recent genetic admixture (e.g., 80 kya) between groups [48, 56–59], with evidence of 2–4% of nuclear DNA in Eurasians that can be traced to Neanderthals [48, 58]. This assumption is likely ubiquitous in a number of Alpha-HPV variants (Fig 7, Table 5), although their pathogenesis, evolution, and epidemiology warrant further study.

Recent evidence indicates that Neanderthals spread out over the Eurasian continent and also admixed with ancestors of the present-day East Asian population [60, 61]. Since HPV16 A4 lineage is exclusively found in East Asians (approximately 40% of HPV16) and presents a higher risk of cervix cancers in Asian populations [62, 63], we speculate that a subset of Neanderthals heading east into Asia over more than 100 thousand years of existence in Eurasia could have interbred with East Asian modern humans and transmitted the HPV16 A4 sublineage and introgressed specific gene alleles that provided a selective advantage to the HPV variants coevolving with them [59, 64]. Overall, HPV16 BCD variants have higher genomic diversity than A isolates (see S4 Table), which may imply a potential population bottleneck of horizontal transmission reducing the diversity of current day A lineage isolates. In contrast, BCD variants have accumulated more genetic mutations, consistent with the observations that African populations and their pathogens have deeper origins reflected in greater diversity [65]. This idea supports one theory that both HPV16 BCD and modern humans arose in Africa (Fig 8). Following a relatively recent *out-of-Africa* migration, the modern humans acquired the A variant from sex with archaic hominins and possibly carried D variants into Eurasia under conditions of a small population size. The ancestors of East Asian people crossed the Bering Strait and were early populators of the Americas (based on historical records and genetic relatedness) [66]. Surprisingly, the D lineage is phylogenetically rooted in the African clade, but we did not find a major reservoir of the D lineage in the present-day African populations. This interesting observation suggests either an advantage of niche colonization and expansion of HPV16 D variants in Native Americans or a bottleneck of HPV16 variants present in people populating the Americans. Alternatively, the lack of A4 and the high proportion of D lineages in the Americans could be the result of an early colonization of the Americas by an unknown group from Africa. More data is needed to sort out the evolutionary history of the HPV16 D lineage and might provide clues to new features of the populating of the Americas.

Sexual interactions between archaic hominins and modern human ancestors likely occurred over multiple time- and space-scales. For example, viral transmission might have also occurred from modern humans to Neanderthals/Denisovans, based on the evidence of ancient gene flow from early modern humans into Eastern Neanderthals [57]. Since PVs usually establish infections at the basal layer of epithelial cells, it will be impossible to detect viruses from fossil bones of archaic hominins and document the presence of HPVs in archaic hominin populations [20]. The evolutionary histories and origins of modern *H*. *sapiens* are undergoing dramatic revisions with the introduction of advanced sequencing techniques and methods to analyze genomic samples from archaic hominin specimens [67–69]. Since the reproductive success per copulation between *H*. *sapiens* and archaic hominins is predicted to have lower viability than that of modern human reproductive events, high levels of sexual interaction were likely present facilitating HPV transmission, in addition to genetic introgression observed in modern non-African populations [70]. For example, the human leukocyte antigen (HLA) B*07:02 and C*07:02 alleles associated with increased risk in cervix cancers appear to be introgressed regions in present-day Eurasians and Melanesians from Neanderthals or Denisovans [71–73]. This also suggests that adaptive introgression of modern humans from archaic hominins influences the pathogenic outcome of these infections by as yet unknown mechanisms [70, 74]. However, it can be speculated that introgressed genes providing some selective advantage to hybrid human-archaic hominin offsprings could also make them more susceptible to HPV variants adapted to archaic hominins over hundreds of thousands of years of coevolution. The introgressed genes are most likely related to immunity against infections, whatever the pathogens might be and HPV was along for the ride, since HPV is not known to affect reproductive fitness of the host.

This study has its strengths and limitations. We expand the current understanding of HPV16 evolution beyond the recent description of HPV transmission between archaic and modern humans that used existing data [20] in important ways. We have expanded the understanding of HPV16 in the context of human and non-human primate PV evolution by characterizing additional New World and Old World monkey PVs and using the known divergence times of specific primate species to establish a valid molecular clock. This approach was used to establish the times of Neanderthal divergences [48]. We demonstrate that niche adaption had to proceed viral-host coevolution, and suggest that subsequent niche adaptation might underlie the difference in prevalence of HPV16 and HPV18 in cervical squamous and glandular lesions. We have identified and characterized additional HPV16 variants enabling us to establish the HPV16 variant taxonomy that includes subvariants that have unique biological characteristics [53]. Moreover, we propose that evolution of HPV16 A in Neanderthals over time led to allopatric emergence of the HPV16 A4 lineage as Neanderthals moved east and interbred with modern humans in Asia. We have also expanded the number of HPV16 isolates from around the world to establish the global distribution of HPV16 variants. Lastly, we provide new interpretations and questions on the HPV16 D lineage that is part of the African clade, but is highly prevalent in South/Central America. Nevertheless, there are also limitations to the current study and interpretations. The understanding of human evolution is constantly being challenged with new data and it is possible the models of human evolution used in this study will change [75]. We have not sampled every population and it is possible that additional HPV16 isolate data could change our interpretations. The data obtained on the geographic locations of the HPV partial sequences could be incorrect resulting in underestimating the true associations between variants and historic origins. Lastly, it is possible that very low population sizes of humans migrating out of Africa carried HPV16 A lineage variants leaving no traces in Africa, but expanding throughout Eurasia. This unlikely possibility would influence the interpretations of both our work and that of previous studies analyzing the evolution of HPV16 [20].

In conclusion, the biology and natural life cycle of oncogenic HPVs that results in infectious viral particles (i.e., vegetative virus life cycle) is highly adapted to the differentiation program of epithelial cells [76]. Poorly differentiated precancerous and cancerous cells in the cervix do not support the HPV vegetative life cycle, and thus viral-associated transformation does not contribute to the fitness of HPVs. Viral phenotypes that serve to adapt to a specific ecological niche, evade host immune mechanisms, and support persistent viral production, however, should contribute to viral fitness. Therefore, further investigations of viral-host interactions and the underlying mechanisms of viral oncogenicity, should continue to focus on features of viral evolution and niche adaptation that contribute to fitness, since the oncogenic outcome of HPV infections appear to be “collateral damage” affecting host morbidity and mortality. The current data provides a framework to unravel the mysteries of oncogenic HPV genomes as we expand our understanding of viral-host evolution.

## Materials and methods

### Ethics statement

The studies providing human cellular samples have been approved by the Institution Review Board of the Albert Einstein College of Medicine, Bronx, NY, and the Joint Chinese University of Hong Kong-New Territories East Cluster Clinical Research Ethics Committee. All human subjects were older than 18 years of age and samples were anonymized without individual identifying information. Written informed consent was obtained from each participant.

The animal use protocol was reviewed and approved by the Institutional Animal Care and Use Committee (IACUC) of Albert Einstein College of Medicine (protocol number 20060908). All procedures involving animals were conducted in compliance with applicable state and federal laws, guidelines established by the Animal Care and Use Committees of the respective institutions, and standards of the U.S. Department of Health and Human Services, including the National Institutes of Health Guide for the Care and Use of Laboratory Animals. The programs for animal care and welfare at Albert Einstein College of Medicine has been fully accredited by the Association for Assessment and Accreditation of Laboratory Animal Care (AAALAC). The Animal Welfare Assurance (A3312-01) is on file with the Office for Laboratory Animal Welfare.

### *Saimiri sciureus* and *Macaca mulatta* papillomavirus isolates and complete genome characterization

The *Saimiri sciureus* PV DNA was isolated from exfoliated cervical cells of two adult female squirrel monkeys screened using polymerase chain reaction (PCR)-based MY09/11 and FAP59/64 primer systems [77, 78]. Sequences from the PCR products were compared with a PV database maintained in the Burk lab using a Blastn search and shown to have < 90% similarities to previously characterized PV types. The whole genomes were PCR-amplified as two overlapping fragments using degenerate primer sets designed on available L1 gene sequences and consensus E1 alignments, and subsequently Sanger sequenced using primer walking in the Einstein Sequencing Facility, New York [33]. Geneious R9.1.7 was used to assemble segmented sequences into the complete genome sequences and identify ORFs [79].

The *Macaca mulatta* PV DNA was purified from exfoliated cervical cells of one adult female rhesus monkey and swabs from the penis surface of one adult male rhesus monkey. The viral DNA was initially detected using multiplexed next-generation sequencing (NGS) assays targeting two small fragments (136 bp and 83 bp, respectively) within the L1 ORF [80, 81]. Sequences of a Blastn search against a PV database showed < 90% similarities to characterized PV types. The total DNA underwent a metagenomic sequencing on an Illumina HiSeq4000 at Weill Cornell Medicine Genomics Resources Core Facility, New York, using paired-end 100 bp reads. The short reads were filtered for host genome contamination and assembled *de novo* using Megahit v1.0.6 to build contigs with long length [82]. The whole genomes of novel *Macaca mulatta* PVs were validated using type-specific PCR in three overlapping fragments and Sanger sequencing using a primer walking strategy.

The complete genome sequences of SscPV1/2/3 and MmPV2/3/4 have been submitted to NCBI/GenBank database, with access numbers of JF304765 to JF304767 and MG837557 to MG837559, respectively.

### Human papillomavirus type 16 complete genome sequencing

In our previous work, we sequenced the complete genomes of 78 HPV16 isolates (see HPV16 list in S3 Table) [83, 84]. In the current study, 122 cervicovaginal samples containing HPV16 DNA were randomly chosen from the Kaiser Permanente Northern California (KPNC)-NCI HPV Persistence and Progression (PaP) cohort study [85] and a population-based HPV prevalence survey coordinated by the International Agency for Research on Cancer (IARC) [63]. The complete genomes were characterized using nested overlapping PCR and Sanger sequencing as previously reported [86]. The PaP study samples were also sequenced using Ion PGM platform [87]. In addition, 12 HPV16 complete genomes sequenced by others were included in this study [88–92].

### Phylogenetic analyses and tree construction

To evaluate the phylogenetic relationships of PVs, the concatenated nucleotide sequences of four open reading frames (ORFs) of the E1, E2, L2, and L1 genes of 141 PV types representing 132 species and unique hosts were used (see PV list in S2 Table, column labelled “Selected type” marked yes). Because all known PVs contain these four core ORFs, the concatenated partitions provide a comprehensive evaluation of the evolutionary history of *Papillomaviridae*. In addition, the highly conserved E1 early gene and L1 late gene were used to characterize phylogenetic incongruence. The nucleotide sequences of each coding region were aligned based on the corresponding amino acid sequences previously aligned using MUSCLE v3.8.31 [93] in Geneious R9.1.7. For HPV16 lineage/sublineage classification and phylogenetic analyses, all 212 complete genome nucleotide sequences (see HPV16 list in S3 Table) were linearized at the ATG of the E1 ORF and aligned using MAFFT v7.221 [94].

Maximum likelihood (ML) trees were constructed using RAxML MPI v8.2.3 [95] and PhyML MPI v3.1 [96] with optimized parameters based on the aligned complete genome nucleotide sequences. Data were bootstrap resampled 1,000 times in RAxML and PhyML. MrBayes v3.1.2 [97] with 10,000,000 cycles for the Markov chain Monte Carlo (MCMC) algorithm was used to generate Bayesian trees. A 10% discarded burn-in was set to eliminate iterations at the beginning of the MCMC run. The average standard deviation of split frequencies was checked to confirm the independent analyses approach stationarity when the convergence diagnostic approached <0.001 as runs converge. For Bayesian tree construction, the computer program ModelTest v3.7 [98] was used to identify the best evolutionary model; the identified General Time Reversible (GTR) model was set for among-site rate variation and allowed substitution rates of aligned sequences to be different. The CIPRES Science Gateway [99] was accessed to facilitate RAxML and MrBayes high-performance computation.

Permutational multivariate analysis of variance was performed using the *adonis* function in R’s package ‘*vegan*’ and the pairwise distance based on 220 primate papillomavirus E1-E2-L2-L1 nucleotide sequences (S2 Table).

### Geographic dispersal of HPV16 variants worldwide

A dataset of 3256 partial sequences spanning variable genes/regions of HPV16 was obtained from GenBank that included the geographic source of the sequences mainly from indigenous ethnicities and/or local communities including 22 countries/regions throughout the world. These included, in Africa: Burkina Faso [100], Nigeria [101], Rwanda [102], Uganda [103], and Zambia [104]; in Asia: China [105–107], India [108, 109], Japan [110], Korea [111], and Thailand [112, 113]; in Europe: Germany [114], Italy [115–118], Netherland [119, 120], Portugal [121], Russian [122], Spain [123], and United Kingdom [124]; in North America: Canada (GenBank, see details in Table 3), Costa Rica [9]; in South/Central America: Bazile [125–127] and Mexico [128–131]; and Australia [132] (see Table 3). We used a maximum phylogenetic likelihood algorithm in pplacer v1.1.alpha17 [133] to place partial sequences on a reference tree inferred from an alignment composed of the 212 HPV16 variant complete genomes described in this study. A cutoff value of maximum likelihood ≥ 0.8 was set as confident assignment of HPV16 isolates into lineages and sublineages. The abundance of each lineage from the same country was combined and normalized using a percentage. According to the geographic patterns of HPV16 variants [44], four ethnical groups, namely African, Asian, Caucasian, and South/Central American, were summarized; for each HPV16 (sub)lineage, its frequency in each group was calculated based on the summary of individual percent abundance divided by the summary of total percent abundance. We used a weighted UniFrac method in R’s package ‘*GUniFrac’* [134] to calculate the pairwise distances between geographic locations, based on which a principle component analysis (PCoA) was performed to visualize the clustering of geographic groups of HPV16 variants using the *betadisper* function in R’s package ‘*vegan*’.

### Estimation of divergence times

We used a Bayesian Markov Chain Monte Carlo (MCMC) method implemented by BEAST v2.4.5 [135] and the previously published PV evolutionary rates [17] to estimate the divergence times of primate PVs from their most recent common ancestors (MRCAs). Times were calculated separately for *Alphapapillomavirus* (n = 85), *Betapapillomavirus* (n = 54), and *Gammapapillomavirus* (n = 81) (S2 Table), given that primate PVs, taken together, do not follow strict virus-host codivergence. Three tree priors were estimated using the following demographic models: (1) coalescent constant population, (2) Yule model, and (3) coalescent Bayesian skyline, with assumptions that (1) the PV genome has a strict mutation rate or (2) there is an uncorrelated lognormal distribution (UCLD) molecular clock model of rate variation among branches, resulting in six combinations of models. In addition, we chose the GTR sequence revolution model with the gamma-distributed rate heterogeneity among sites and a proportion of invariant sites (GTR + G + I) determined by the best-fit model approach of Modeltest v3.7 [98]. The concatenated nucleotide sequence partitions of six ORFs (E6, E7, E1, E2, L2, and L1) with variable rates of substitution over time were used: 2.39 × 10^−8^ (95% confidence interval 1.70–3.26 × 10^−8^) substitutions per site per year for the E6 gene, 1.44 × 10^−8^ (0.97–2.00 × 10^−8^) for the E7 gene, 1.76 × 10^−8^ (95% CI: 1.20–2.31 × 10^−8^) for the E1 gene, 2.11 × 10^−8^ (95% CI: 1.52–2.81 × 10^−8^) for the E2 gene, 2.13 × 10^−8^ (95% CI: 1.46–2.76 × 10^−8^) for the L2 gene, and 1.84 × 10^−8^ (95% CI: 1.27–2.35 × 10^−8^) for the L1 gene, as previously described [17]. In order to calibrate the divergence times, we introduced three time points inside and at the root of the *Alphapapillomavirus* tree, with assumptions of codivergence histories between primate PVs and their hosts: (1) the node between HPV13 and chimpanzee PpPV1 (*Pan paniscus* PV 1) at 7 mya (95% CI, 6–8 mya) matching the split between hominin and chimpanzee ancestors; (2) the node between the species Alpha-12 (represented by *Macaca mulatta* PV 1) and Alpha-9/11 (represented by HPV16) at 28 mya (25–31 mya) matching the speciation between hominin and macaque ancestors; and (3) the node between *Alphapapillomavirus* and *Dyoomikronpapillomavirus* (represented by *Saimiri sciureus* PV 1) at 49 mya (41–58 mya) matching the divergence between Old World and New World monkey ancestors [26]. For *Betapapillomavirus* and *Gammapapillomavirus* trees, the calibration time point(s) was set between macaque PVs and their closet HPV relatives.

To estimate divergence times of HPV16 complete genome variants, a *Hominin-host-switch* (*HHS*) model assuming there was an ancestral viral transmission between archaic and modern human populations [20] was applied by setting two evolutionary time points to calibrate the HPV16 variant phylogenetic tree: (1) the archaic divergence of modern humans and Neanderthals/Denisovans around 500 thousand years ago (kya) (95% CI, 400–600 kya) [136] matching the split between HPV16 Eurasian (A) and African variants (BCD), and (2) the modern human *out-of-Africa* migration at 90 kya (95% CI, 60–120 kya) [45, 137], locating the era when HPV16 D variants diverged from their most recent common ancestor (MRCA). A HPV16 variant substitution rate was used for validation of a uniform prior rate: 1.84 x 10^−8^ (95% CI, 1.43–2.21 x 10^−8^) [20], with combinations of three tree priors and two clock models as described above. Due to the lack of geographic/ethnic dispersal information of other HPV type variants, we estimated the youngest divergence events splitting from their MRCA using complete genome alignments and HPV16 variant substitution rate without time point calibration.

To compare the population dynamics of HPV16 variants and the modern human host, Bayesian skyline plots were created using BEAST. A total of 311 globally sampled present-day human mitochondrial DNA (mtDNA) sequences, excluding the 1120 bp non-coding D-loop (that evolves at a higher rate) to give an alignment of 15,471 bp in length [138], were analyzed using a strict clock model and a coalescent Bayesian skyline, with an estimated rate of 2.47 x 10^−8^ (95% CI, 2.16–3.16 x 10^−8^) substitutions per site per year [139], as these sequences have been shown to evolve in a roughly clock-like manner [140, 141]. Two evolutionary time points were used to calibrate the modern human mtDNA tree: (1) the age of the MRCA between the maximum distanced modern humans, estimated to be 171,500 ± 50,000 years ago, and (2) the age of the MRCA of the youngest clade that contains both African and non-African lineages, approximately 52,000 ± 27,500 years ago [140].

The MCMC analysis was run for 100,000,000 steps, with subsampling every 10,000 generations. A discarded burn-in of the first 10% steps was set to refine trees and log-files for further analysis. Effective sample sizes (ESS) of all parameters are >300 (*Alphapapillomavirus* tree) and >2000 (HPV variant trees of each type), indicating that all Bayesian chains were well sampled and have converged. Best model estimates were selected using a posterior simulation-based analogue of Akaike's Information Criterion for MCMC samples (AICM) [142], as implemented in Tracer v.1.6. The lower AICM values indicated a better model fit. A consensus tree was inferred using TreeAnnotater v.2.4.5 and visualized using scripts developed in-house in R. The linear model (*lm*) function in R was used to estimate the correlation between sequence diversity and divergence time of HPV types and variants.

## Supporting information

S1 Fig Phylogeny of papillomaviruses inferred from concatenated E1, E2, L2 and L1 genes. A maximum likelihood phylogenetic tree inferred from the concatenated nucleotide sequence alignment of 4 open reading frames (E1-E2-L1-L2) of 141 papillomavirus types representing 132 species (see PV list in S2 Table, column of “Selected type”). The main clades containing the majority of primate papillomavirus species are highlighted in grey.
(TIF)

S2 Fig Phylogenetic incongruence of papillomaviruses. Maximum likelihood phylogenetic trees were inferred from the nucleotide sequence alignment of E1 (left) and L1 ORFs (right) of 141 papillomavirus types representing 132 species (see PV list with hosts in S2 Table). Although phylogenetic incongruence was observed between trees based on individual genes, the classification of the majority of characterized primate PVs largely corresponds to the grouping based on tissue tropism and biological characteristics.The branches represented by non-human primate papillomaviruses are highlighted in red. Non-primate papillomaviruses are collapsed and joined by grey lines (see comprehensive tree in S3 Fig and S4 Fig). The dot sizes are proportional to the bootstrap percentage supports from RAxML.
(TIF)

S3 Fig Phylogeny of papillomaviruses inferred from E1 gene. A maximum likelihood phylogenetic tree inferred from the nucleotide sequence alignment of E1 gene of 141 papillomavirus types representing 132 species (see PV list in S2 Table, column of “Selected type”). The main clades containing the majority of primate papillomavirus species are highlighted in grey.
(TIF)

S4 Fig Phylogeny of papillomaviruses inferred from L1 gene. A maximum likelihood phylogenetic tree inferred from the nucleotide sequence alignment of L1 gene of 141 papillomavirus types representing 132 species (see PV list in S2 Table, column of “Selected types”). The main clades containing the majority of primate papillomavirus species are highlighted in grey.
(TIF)

S5 Fig Divergence time estimation of Alphapapillomaviruses and Dyoomikronpapillomavirus to their most recent common ancestors (MRCAs). A Bayesian MCMC method was used to estimate divergence times as described in the methods. Branch lengths are proportional to divergence times. The branches in red refer to non-human primate papillomaviruses. Numbers above the nodes with circles are the mean estimated divergence times in millions of years (M) between human and non-human papillomavirus clades. The bars in grey represent the 95% highest posterior density (HPD) interval for the divergence times. The viral genomes included can be found in S2 Table.
(TIF)

S6 Fig Divergence time estimation of Betapapillomaviruses to their most recent common ancestors (MRCAs). A Bayesian MCMC method was used to estimate divergence times as described in the methods. Branch lengths are proportional to divergence times. The branches in red refer to non-human primate papillomaviruses. Numbers above the nodes with circles are the mean estimated divergence times in millions of years (M) between human and non-human papillomavirus clades. The bars in grey represent the 95% highest posterior density (HPD) interval for the divergence times. The viral genomes included can be found in S2 Table.
(TIF)

S7 Fig Divergence time estimation of Gammapapillomaviruses to their most recent common ancestors (MRCAs). A Bayesian MCMC method was used to estimate divergence times as described in the methods. Branch lengths are proportional to divergence times. The branches in red refer to non-human primate papillomaviruses. Numbers above the nodes with circles are the mean estimated divergence times in millions of years (M) between human and non-human papillomavirus clades. The bars in grey represent the 95% highest posterior density (HPD) interval for the divergence times. The viral genomes included can be found in S2 Table.
(TIF)

S8 Fig HPV16 complete genome tree topology. Maximum likelihood trees of HPV16 variant isolates inferred from 212 complete genomes listed in S3 Table. Variant lineages (e.g., termed A and B, etc.) and sublineages (e.g., termed A1 and A2, etc.) are named using an alphanumeric nomenclature system. Inter-sublineage bootstrap supports by PhyML and RAxML are labeled at the key nodes. Colors represent different HPV16 lineages. The bar indicates the nucleotide substitution of unit changes per site.
(TIF)

S9 Fig Heatmap of HPV16 complete genome pairwise diversity. Pairwise sequence identity based on the nucleotide sequence alignment of 212 HPV16 complete genomes was measured and represented as a heatmap and scaled such that the maximum inter-sequence identity differences (2.23%) are displayed as red and the minimum inter-sequence identity differences (0.00%) as blue.
(TIF)

S10 Fig Divergence time estimation of HPV16 complete genome variants. A Bayesian MCMC method was used to calculate the divergence times of HPV16 complete genome variants from their most recent common ancestors as described in the methods. A previously published HPV16 variant substitution rate and two human evolutionary time points of calibration (red circles) were set. Branch lengths are proportional to the times and are scaled in millions of years (M). Grey bars indicate the 95% highest posterior density (HPD) for the corresponding divergence age. Colors in branches represent distinct HPV16 variant lineages.
(TIF)

S1 Table Novel papillomavirses isolated from squirrel monkeys (*Saimiri sciureus*) and rhesus monkeys (*Macaca mulatta*). (XLSX)

S2 Table List of papillomavirus types used in this study. (XLSX)

S3 Table List of 212 HPV16 complete genome variants. (XLSX)

S4 Table Nucleotide sequence mean difference (± standard error) of HPV16 complete genome variants between and within lineages/sublineages. (XLSX)
