# Supplementary figures and images for: Niche adaptation and viral transmission of human papillomaviruses from archaic hominins to modern humans
Source: PLoS Pathog. 2018 Nov 1;14(11):e1007352. doi: 10.1371/journal.ppat.1007352 (PMC6211759; doi:10.1371/journal.ppat.1007352)

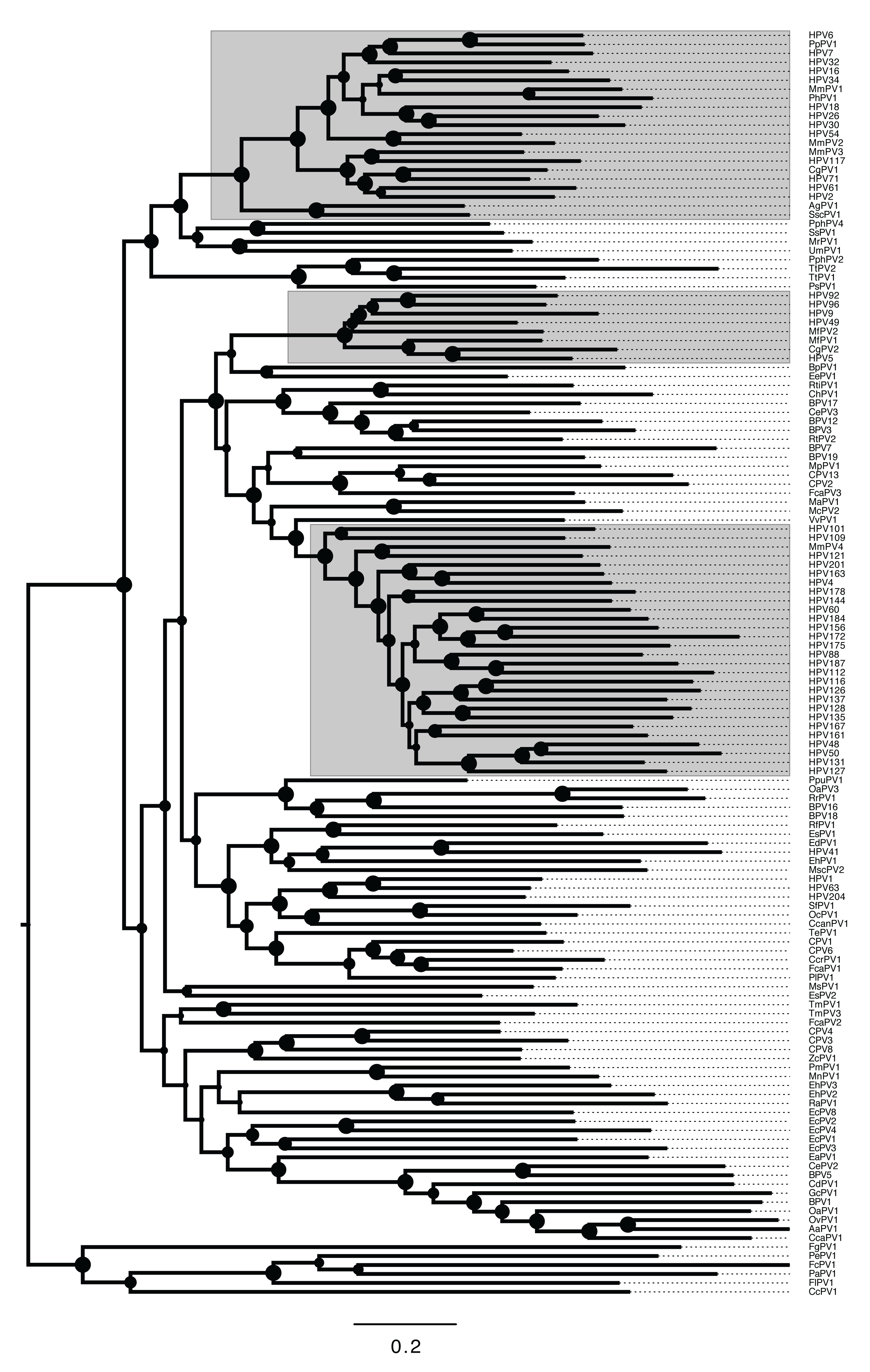

Supplement: S1 Fig — A maximum likelihood phylogenetic tree inferred from the concatenated nucleotide sequence alignment of 4 open reading frames (E1-E2-L1-L2) of 141 papillomavirus types representing 132 species (see PV list in S2 Table, column of “Selected type”). The main clades containing the majority of primate papillomavirus species are highlighted in grey. (TIF) [file ppat.1007352.s001.tif]

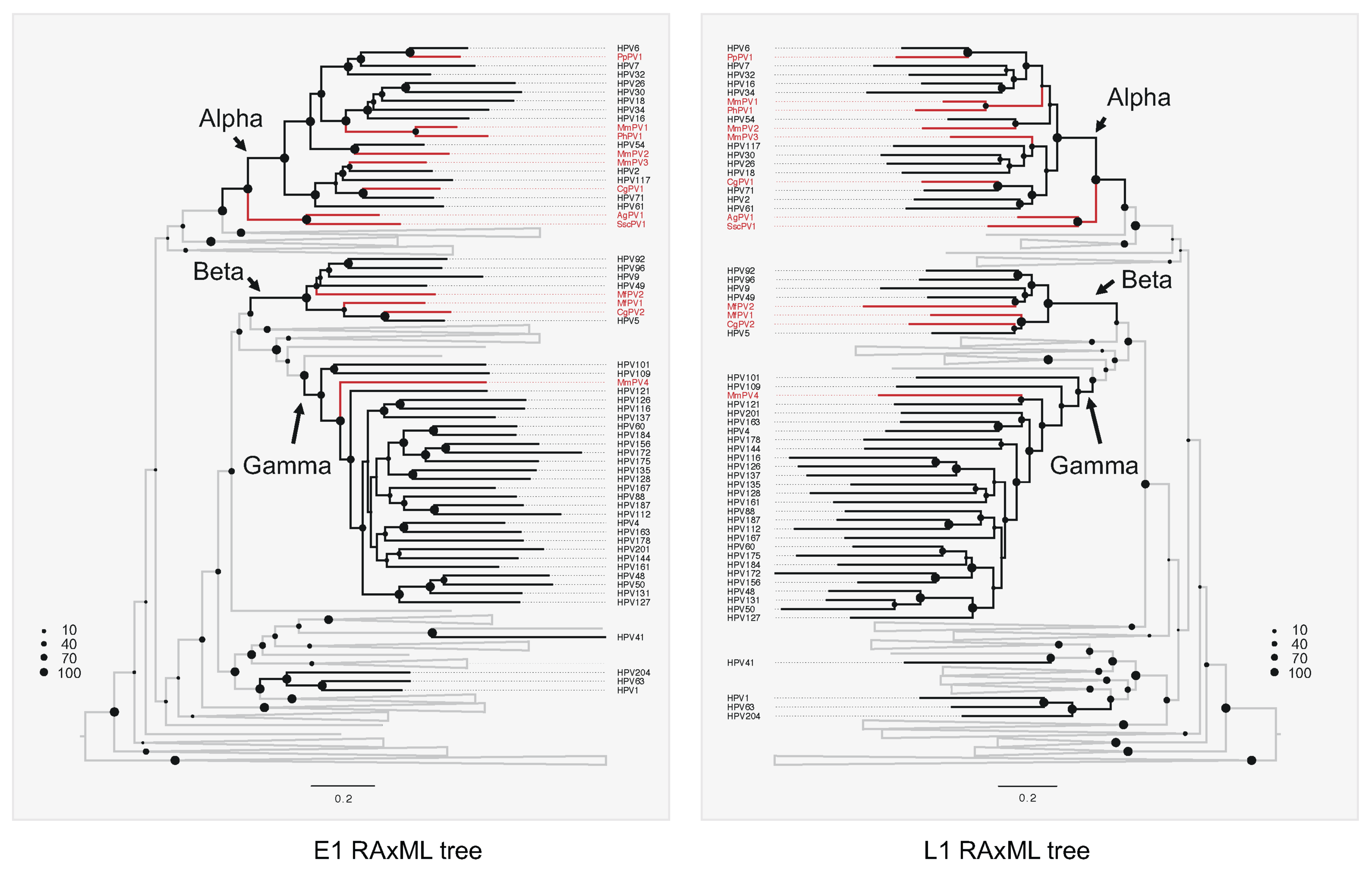

Supplement: S2 Fig — Maximum likelihood phylogenetic trees were inferred from the nucleotide sequence alignment of E1 (left) and L1 ORFs (right) of 141 papillomavirus types representing 132 species (see PV list with hosts in S2 Table). Although phylogenetic incongruence was observed between trees based on individual genes, the classification of the majority of characterized primate PVs largely corresponds to the grouping based on tissue tropism and biological characteristics.The branches represented by non-human primate papillomaviruses are highlighted in red. Non-primate papillomaviruses are collapsed and joined by grey lines (see comprehensive tree in S3 Fig and S4 Fig). The dot sizes are proportional to the bootstrap percentage supports from RAxML. (TIF) [file ppat.1007352.s002.tif]

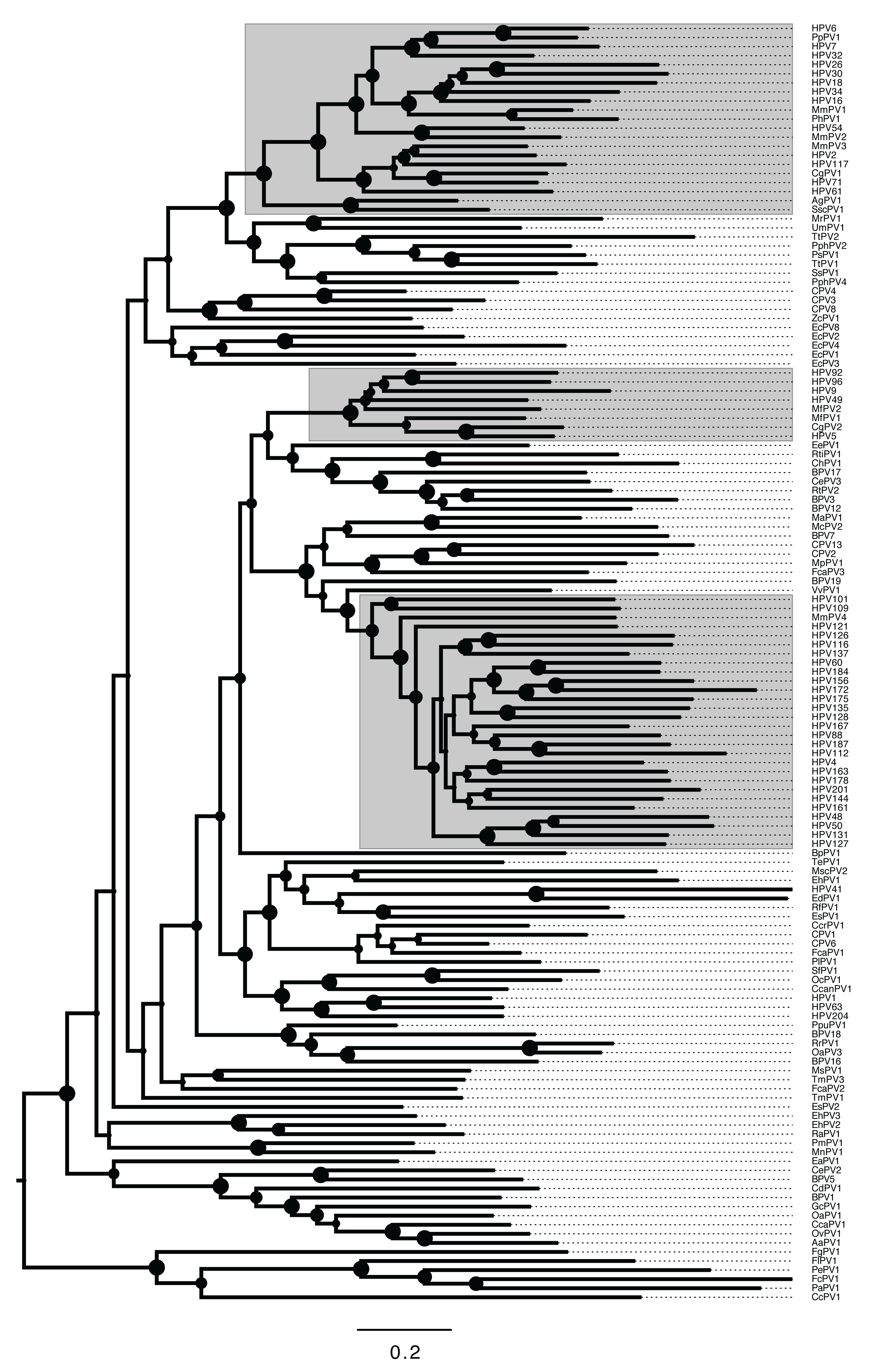

Supplement: S3 Fig — A maximum likelihood phylogenetic tree inferred from the nucleotide sequence alignment of E1 gene of 141 papillomavirus types representing 132 species (see PV list in S2 Table, column of “Selected type”). The main clades containing the majority of primate papillomavirus species are highlighted in grey. (TIF) [file ppat.1007352.s003.tif]

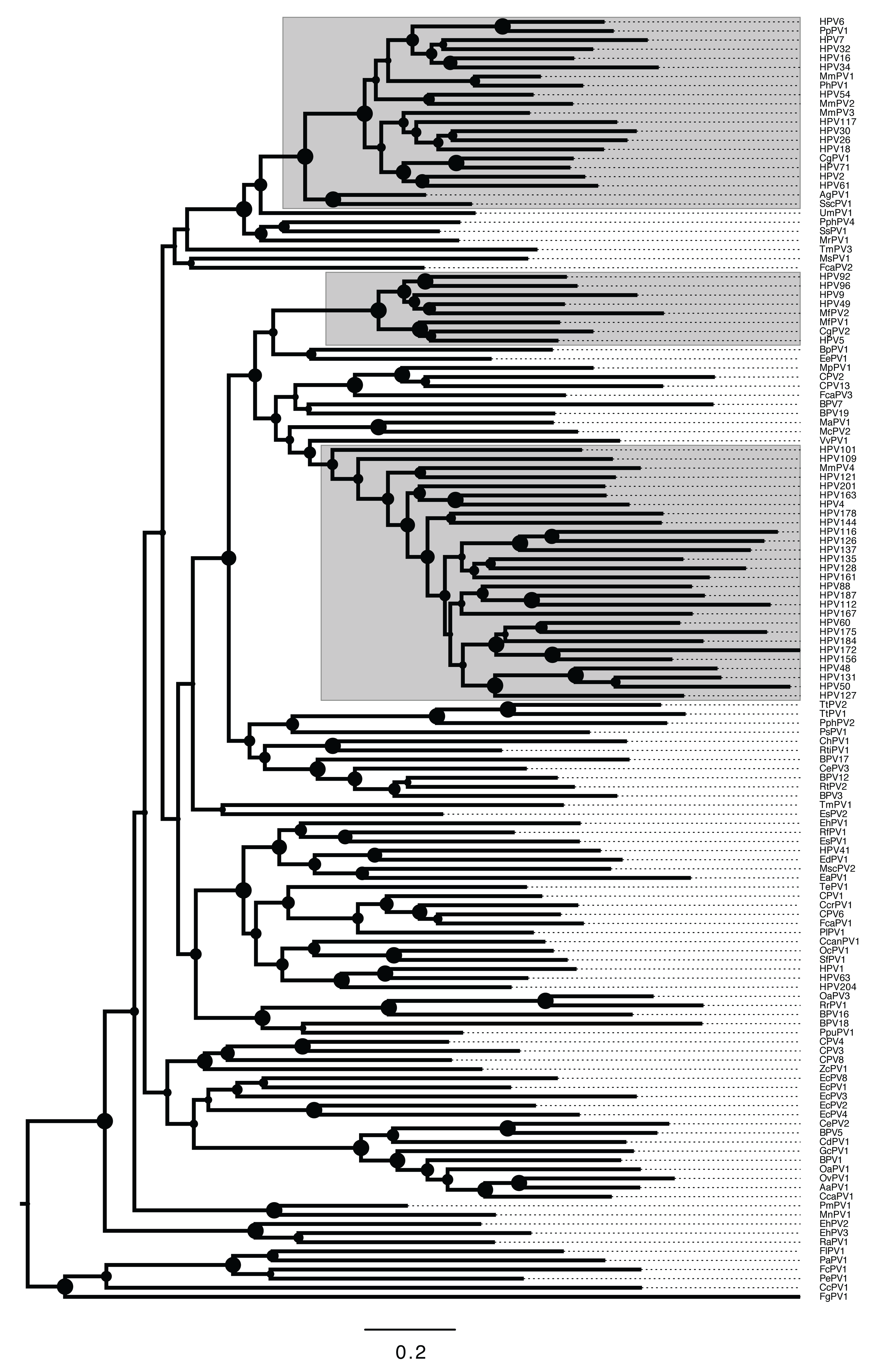

Supplement: S4 Fig — A maximum likelihood phylogenetic tree inferred from the nucleotide sequence alignment of L1 gene of 141 papillomavirus types representing 132 species (see PV list in S2 Table, column of “Selected types”). The main clades containing the majority of primate papillomavirus species are highlighted in grey. (TIF) [file ppat.1007352.s004.tif]

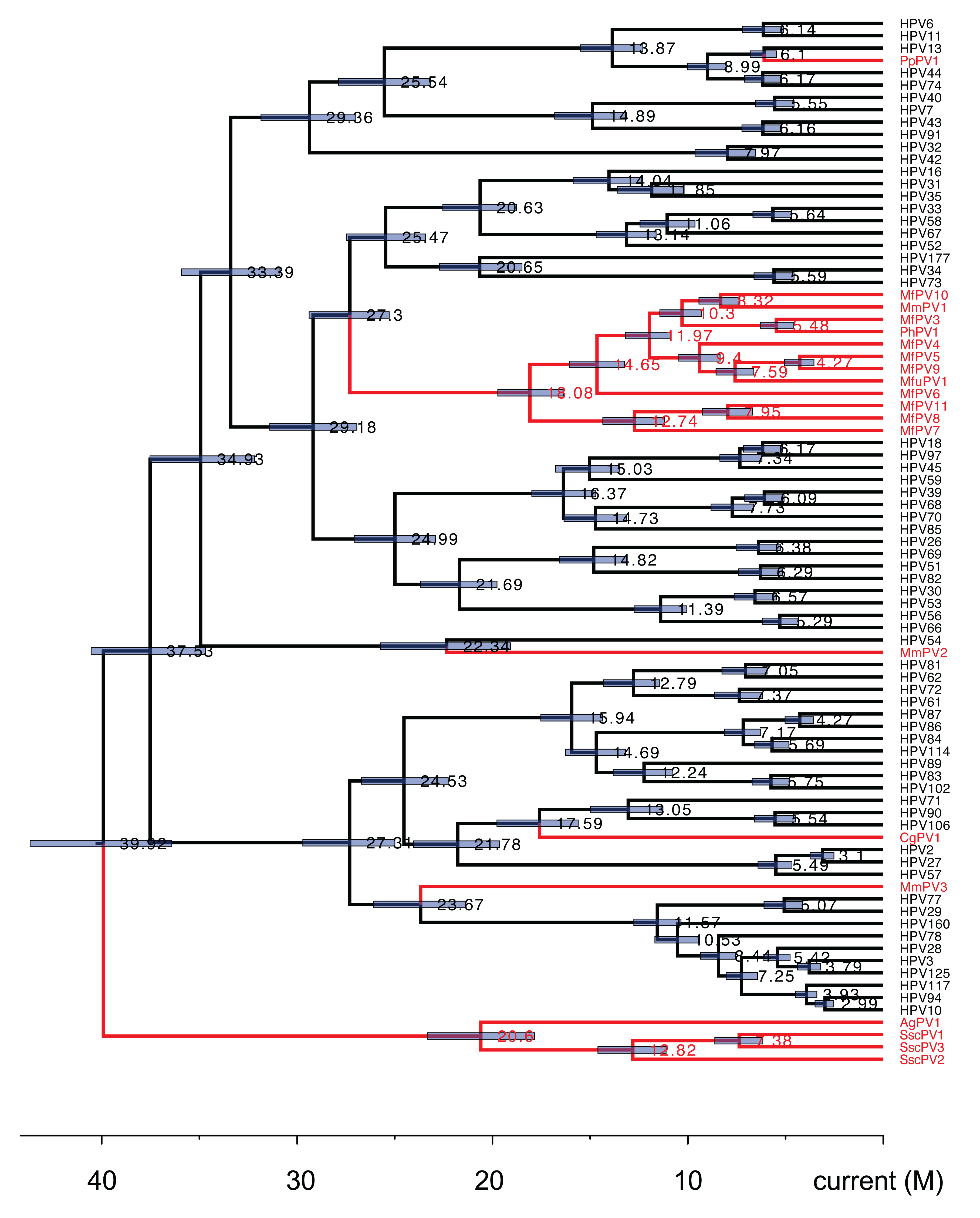

Supplement: S5 Fig — A Bayesian MCMC method was used to estimate divergence times as described in the methods. Branch lengths are proportional to divergence times. The branches in red refer to non-human primate papillomaviruses. Numbers above the nodes with circles are the mean estimated divergence times in millions of years (M) between human and non-human papillomavirus clades. The bars in grey represent the 95% highest posterior density (HPD) interval for the divergence times. The viral genomes included can be found in S2 Table. (TIF) [file ppat.1007352.s005.tif]

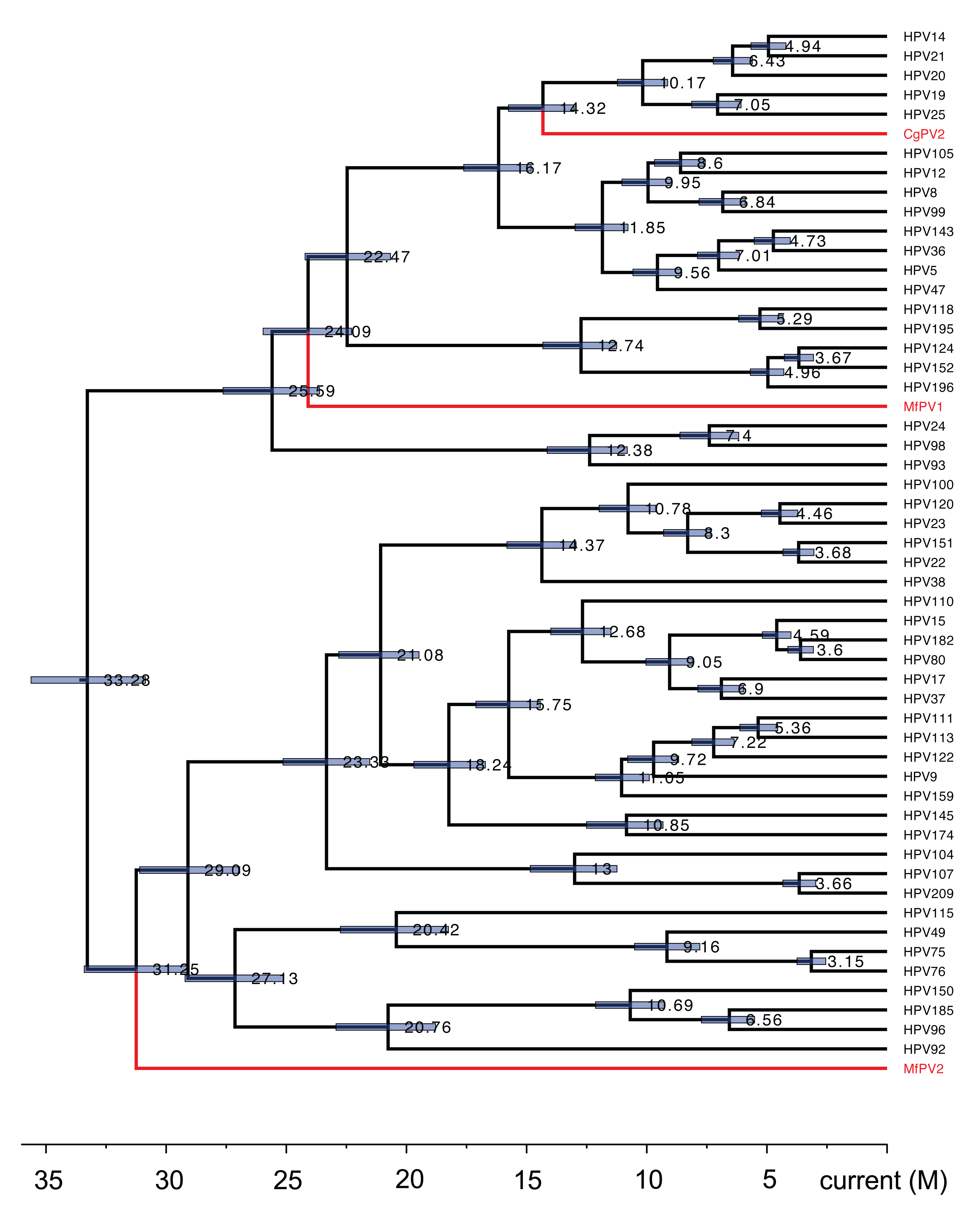

Supplement: S6 Fig — A Bayesian MCMC method was used to estimate divergence times as described in the methods. Branch lengths are proportional to divergence times. The branches in red refer to non-human primate papillomaviruses. Numbers above the nodes with circles are the mean estimated divergence times in millions of years (M) between human and non-human papillomavirus clades. The bars in grey represent the 95% highest posterior density (HPD) interval for the divergence times. The viral genomes included can be found in S2 Table. (TIF) [file ppat.1007352.s006.tif]

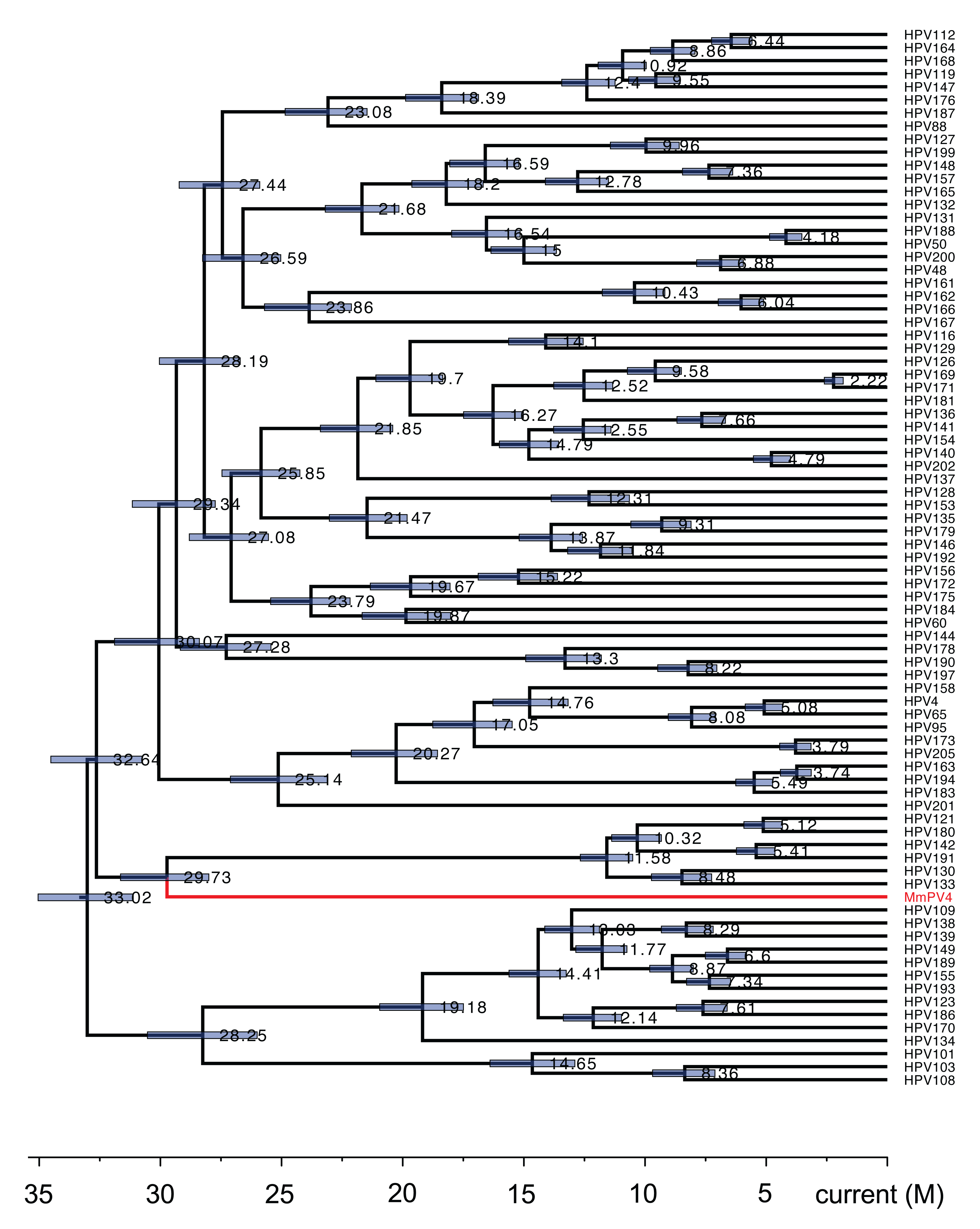

Supplement: S7 Fig — A Bayesian MCMC method was used to estimate divergence times as described in the methods. Branch lengths are proportional to divergence times. The branches in red refer to non-human primate papillomaviruses. Numbers above the nodes with circles are the mean estimated divergence times in millions of years (M) between human and non-human papillomavirus clades. The bars in grey represent the 95% highest posterior density (HPD) interval for the divergence times. The viral genomes included can be found in S2 Table. (TIF) [file ppat.1007352.s007.tif]

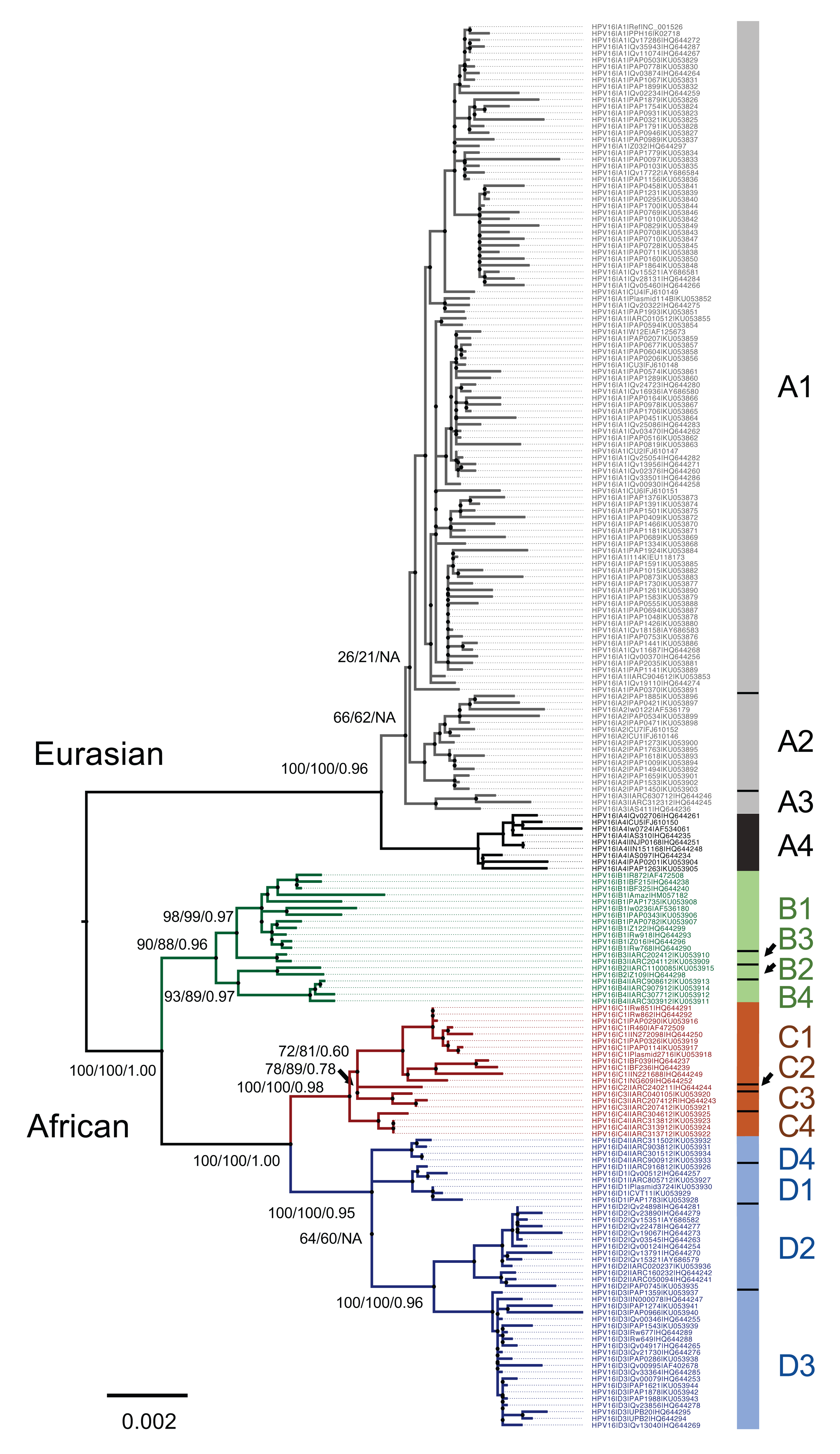

Supplement: S8 Fig — Maximum likelihood trees of HPV16 variant isolates inferred from 212 complete genomes listed in S3 Table. Variant lineages (e.g., termed A and B, etc.) and sublineages (e.g., termed A1 and A2, etc.) are named using an alphanumeric nomenclature system. Inter-sublineage bootstrap supports by PhyML and RAxML are labeled at the key nodes. Colors represent different HPV16 lineages. The bar indicates the nucleotide substitution of unit changes per site. (TIF) [file ppat.1007352.s008.tif]

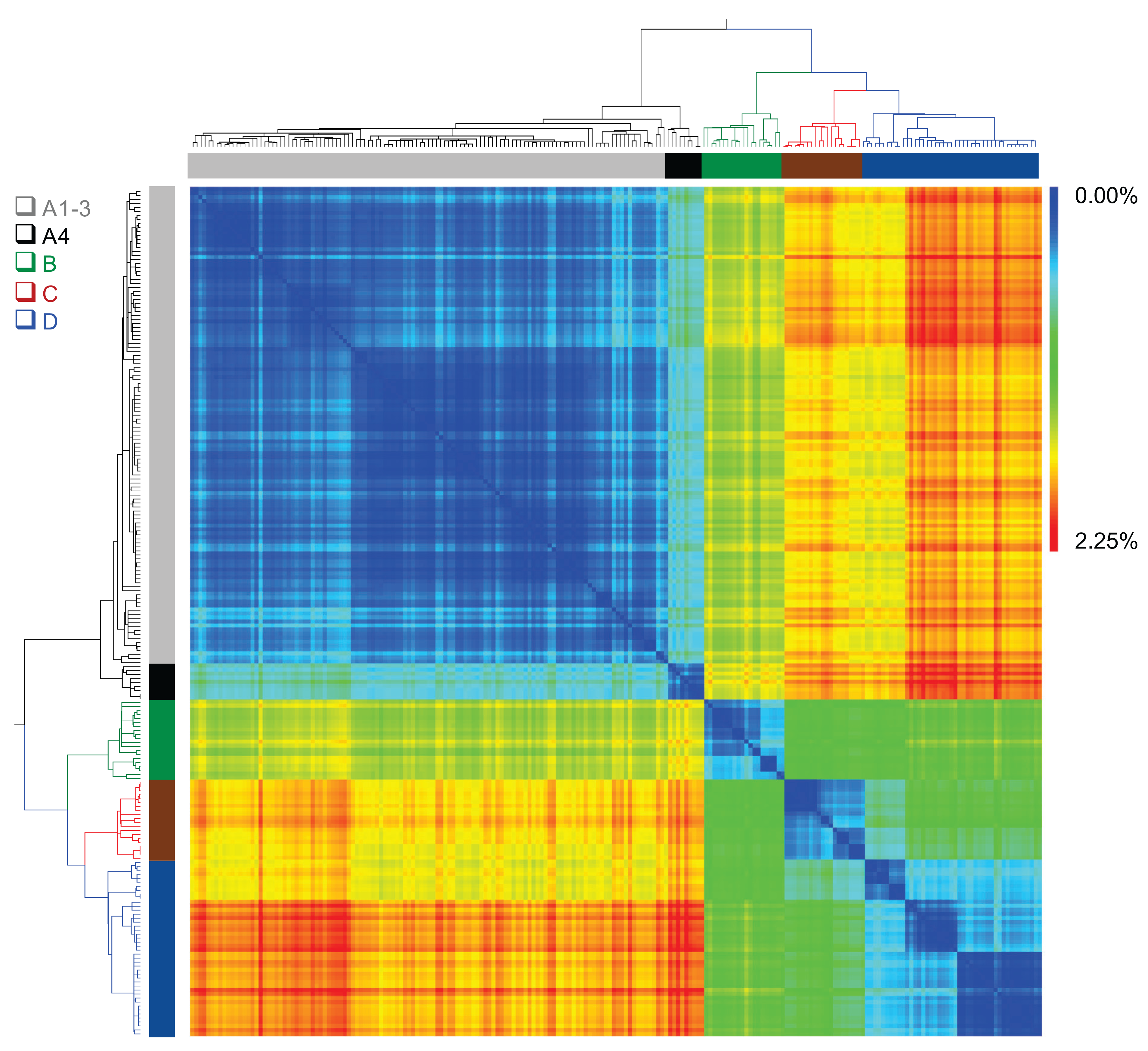

Supplement: S9 Fig — Pairwise sequence identity based on the nucleotide sequence alignment of 212 HPV16 complete genomes was measured and represented as a heatmap and scaled such that the maximum inter-sequence identity differences (2.23%) are displayed as red and the minimum inter-sequence identity differences (0.00%) as blue. (TIF) [file ppat.1007352.s009.tif]

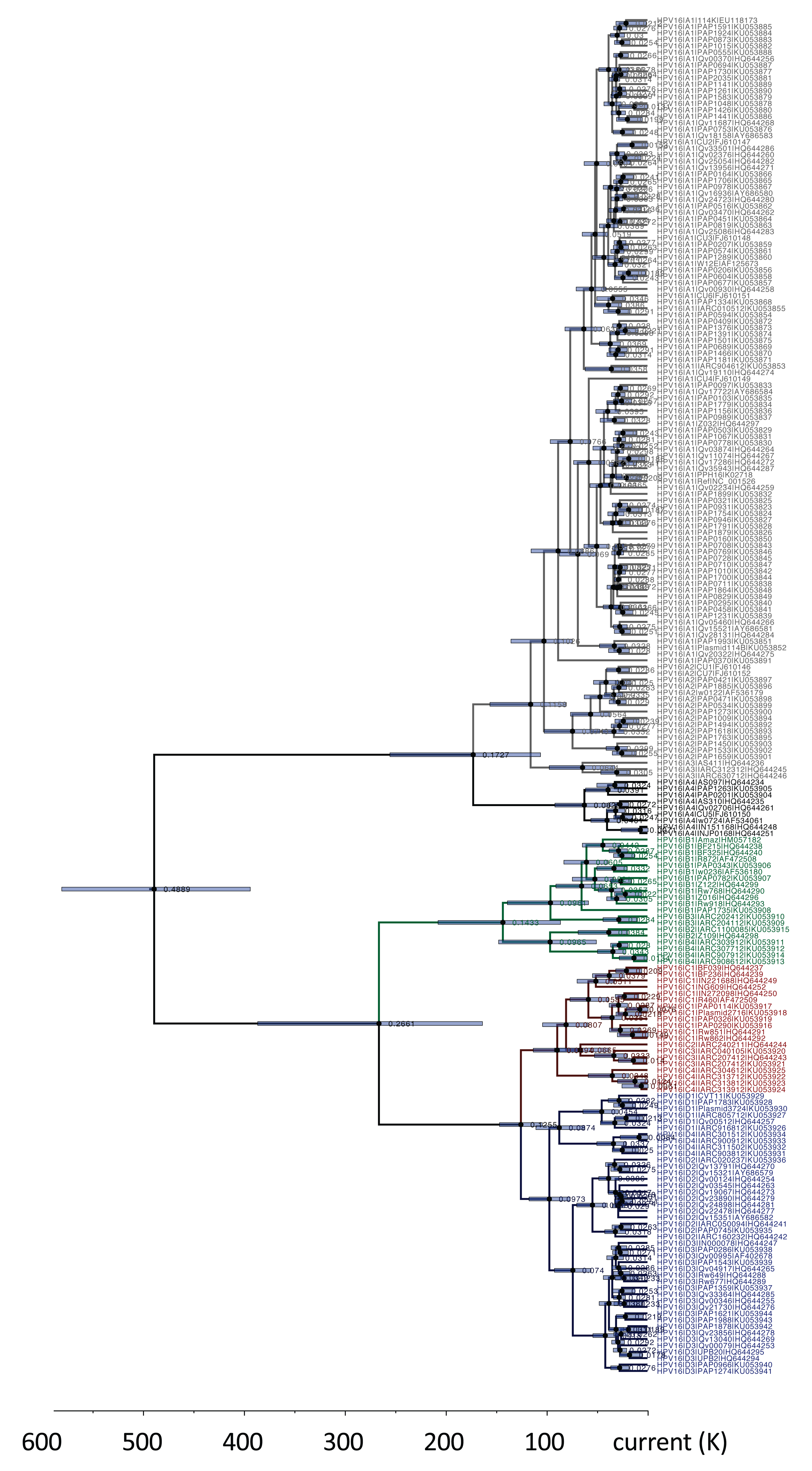

Supplement: S10 Fig — A Bayesian MCMC method was used to calculate the divergence times of HPV16 complete genome variants from their most recent common ancestors as described in the methods. A previously published HPV16 variant substitution rate and two human evolutionary time points of calibration (red circles) were set. Branch lengths are proportional to the times and are scaled in millions of years (M). Grey bars indicate the 95% highest posterior density (HPD) for the corresponding divergence age. Colors in branches represent distinct HPV16 variant lineages. (TIF) [file ppat.1007352.s010.tif]
